# Temporal trends in methane emissions from a small eutrophic reservoir: the key role of a spring burst

**DOI:** 10.5194/bg-18-5291-2021

**Published:** 2021-09-30

**Authors:** Sarah Waldo, Jake J. Beaulieu, William Barnett, D. Adam Balz, Michael J. Vanni, Tanner Williamson, John T. Walker

**Affiliations:** 1 Center for Environmental Measurements and Modeling, Office of Research and Development, United States Environmental Protection Agency, Cincinnati, OH 45268, USA; 2 Neptune and Company, Inc., Lakewood, CO 80215, USA; 3 Pegasus Technical Services, Cincinnati, OH 45268, USA; 4 Department of Biology, Miami University, Oxford, OH 45056, USA; 5 Office of Research and Development, Center for Environmental Measurements and Modeling, United States Environmental Protection Agency, Durham, NC 27709, USA; a currently at: United States Environmental Protection Agency, Region 10, Seattle, WA 98101, USA; b currently at: Office of Research and Development, Center for Environmental Solutions & Emergency Response, United States Environmental Protection Agency, Cincinnati, OH 45268, USA

## Abstract

Waters impounded behind dams (i.e., reservoirs) are important sources of greenhouses gases (GHGs), especially methane (CH_4_), but emission estimates are not well constrained due to high spatial and temporal variability, limitations in monitoring methods to characterize hot spot and hot moment emissions, and the limited number of studies that investigate diurnal, seasonal, and interannual patterns in emissions. In this study, we investigate the temporal patterns and biophysical drivers of CH_4_ emissions from Acton Lake, a small eutrophic reservoir, using a combination of methods: eddy covariance monitoring, continuous warm-season ebullition measurements, spatial emission surveys, and measurements of key drivers of CH_4_ production and emission. We used an artificial neural network to gap fill the eddy covariance time series and to explore the relative importance of biophysical drivers on the interannual timescale. We combined spatial and temporal monitoring information to estimate annual whole-reservoir emissions. Acton Lake had cumulative areal emission rates of 45.6 ± 8.3 and 51.4 ± 4.3 g CH_4_ m^−2^ in 2017 and 2018, respectively, or 109 ± 14 and 123 ± 10 Mg CH_4_ in 2017 and 2018 across the whole 2.4 km^2^ area of the lake. The main difference between years was a period of elevated emissions lasting less than 2 weeks in the spring of 2018, which contributed 17 % of the annual emissions in the shallow region of the reservoir. The spring burst coincided with a phytoplankton bloom, which was likely driven by favorable precipitation and temperature conditions in 2018 compared to 2017. Combining spatially extensive measurements with temporally continuous monitoring enabled us to quantify aspects of the spatial and temporal variability in CH_4_ emission. We found that the relationships between CH_4_ emissions and sediment temperature depended on location within the reservoir, and we observed a clear spatiotemporal offset in maximum CH_4_ emissions as a function of reservoir depth. These findings suggest a strong spatial pattern in CH_4_ biogeochemistry within this relatively small (2.4 km^2^) reservoir. In addressing the need for a better understanding of GHG emissions from reservoirs, there is a trade-off in intensive measurements of one water body vs. short-term and/or spatially limited measurements in many water bodies. The insights from multi-year, continuous, spatially extensive studies like this one can be used to inform both the study design and emission upscaling from spatially or temporally limited results, specifically the importance of trophic status and intra-reservoir variability in assumptions about upscaling CH_4_ emissions.

## 1 Introduction

Reservoirs are a globally important source of methane (CH_4_) and other greenhouse gases (GHGs) to the atmosphere, with recent estimates attributing 773 Tg carbon dioxide equivalent (CO_2_ e) per year to reservoir surface emissions, nearly 80 % (607 Tg CO_2_ e yr^−1^) as CH_4_ (Deemer et al., 2016). This is roughly half the global CH_4_ burden from rice cultivation, estimated as 1100–1360 Tg CO_2_ e yr^−1^ (Ciais et al., 2013). The dominance of CH_4_ in reservoir GHG budgets is due to the combination of gross CH_4_ emissions and methane’s large warming potential relative to CO_2_. Inland waters (lakes, rivers, and reservoirs) can be hot spots for the decomposition of organic matter, and respiration from these waters globally may offset the terrestrial carbon sink by up to 60 % (Cole et al., 2007; Ciais et al., 2013). The carbon dynamics of reservoirs are of special interest for several reasons. Reservoirs generally receive more sediment input (hence organic C) from their watershed than comparable lakes as they tend to be located lower in the landscape and have a larger ratio of catchment area to surface area (Hayes et al., 2017). Reservoirs also tend to drain watersheds with more agricultural or urban land use than the natural lake watersheds (Thornton et al., 1990). The distribution of lakes and reservoirs across the United States is such that in many parts of the country total lentic surface area is dominated by reservoirs. Furthermore, emissions from reservoirs are considered anthropogenic and thus should be included in national GHG emission inventories reported to the United Nations (Lovelock et al., 2019).

Emissions of GHGs from reservoirs are highly variable in space and time, making reservoir GHG budgets difficult to constrain. This is especially true for CH_4_, the production and emission pathways of which are highly dynamic. One key production pathway of CH_4_ in water bodies is methanogenesis in anoxic sediment. Some of this CH_4_ dissolves into the water column where it may be oxidized into CO_2_ by methanotrophs or may diffuse to the atmosphere. Methane may also accumulate as bubbles in the sediment until the buoyant force of the gas bubble overcomes the overlying static pressure. The rate of this CH_4_ bubbling, or ebullition, is affected by several biological and physical factors including carbon substrate availability, sediment temperature, oxygen availability, turbulence, and overlying pressure (Tuser et al., 2017). Thus, ebullition is highly variable in space and time (Wik et al., 2016). Another potentially important source of CH_4_ is production in oxic surface water, considered a “paradox” until recently (Schmidt and Conrad 1993; Grossart et al., 2011; Tang et al., 2014, 2016; DelSontro et al., 2018b). The rate of diffusive flux from surface waters can be highly dynamic as it depends on the balance between production and emission (Hartmann et al., 2020).

Although the body of knowledge on CH_4_ emissions from inland waters has grown considerably over the past decades, the high degree of spatial and temporal variability in emissions, coupled with limitations in monitoring methods, mean that many questions about reservoir emission behavior remain. Recent studies have highlighted the importance of interannual patterns (Room et al., 2014), seasonal patterns (Yvon-Durocher et al., 2014), diurnal patterns (Podgrajsek et al., 2014; Deshmukh et al., 2014), sub-daily pulse events (Zhang et al., 2021), lake-zone spatial patterns (Juutienen et al., 2009; DelSontro et al., 2011; Maeck et al., 2013; McClure et al., 2020), and the relative contributions of hot spots (Wik et al., 2016; Beaulieu et al., 2016), hot moments (Bastien et al., 2011; Demarty et al., 2011; Jammet et al., 2015; Beaulieu et al., 2018; Harrison et al., 2018), and food web dynamics (Bartosiewicz et al., 2021; Grasset et al., 2018) in accurately characterizing lake and reservoir CH_4_ emissions. Under-sampling in irregular systems leads to underestimation (Wik et al., 2016). The synthesis by Deemer et al. (2016) showed that reservoir GHG emission studies using spatially integrated methods reported higher $F_{{\text{CH}}_{4}}$ than studies using survey methods. Despite the need to better capture the spatiotemporal dynamics of reservoir CH_4_ fluxes $F_{{\text{CH}}_{4}}$ and its drivers, most monitoring studies to date have used survey methods that are often short-term, intermittent, and/or spatially limited.

Use of micrometeorological methods such as eddy covariance (EC) to monitor reservoir $F_{{\text{CH}}_{4}}$ can address many of the monitoring challenges by providing pseudo-continuous, long-term, spatially integrated flux measurements. A low-power open-path CH_4_ sensor capable of making measurements for EC has only been available since circa 2011 (McDermitt et al., 2011), and using micrometeorological techniques to measure fluxes over open water (vs. land) can be difficult due to siting, footprint, and boundary layer turbulence considerations (Kenny et al., 2017; Higgins et al., 2013; Sahlee et al., 2014). Thus, relatively few studies have used EC to characterize $F_{{\text{CH}}_{4}}$ over inland waters (Jammet et al., 2015, 2017; Deshmukh et al., 2014; Eugster et al., 2011; Schubert at al., 2012; Podgrajsek et al., 2014a, b; Beaulieu et al., 2018). Further highlighting the scarcity of studies using this technique, the recent FLUXNET-CH_4_ synthesis (Knox et al., 2019) of long-term (*>* 1 year) EC monitoring of $F_{{\text{CH}}_{4}}$ had only two open-water sites among the 60 included. To our knowledge, this study is only the second to report pseudo-continuous, multi-year $F_{{\text{CH}}_{4}}$ results over open water, and the first to report long-term $F_{{\text{CH}}_{4}}$ over open water in a temperate region, for a eutrophic system, and for a reservoir.

This study reports the results of 2 years of pseudo-continuous (via EC and active funnel traps for ebullition), spatially extensive (via spatially balanced CH_4_ emission surveys) measurements of $F_{{\text{CH}}_{4}}$ and key drivers of CH_4_ production and emission. We organize our findings around two questions that can inform both the design of future monitoring studies and emission upscaling from limited results: (1) How important can interannual and intra-lake variability be in a single reservoir, and what causes it? (2) What does this tell us about how limited monitoring resources can best be used to constrain reservoir methane emissions?

## 2 Methods

### 2.1 Site description

**Figure 1.**
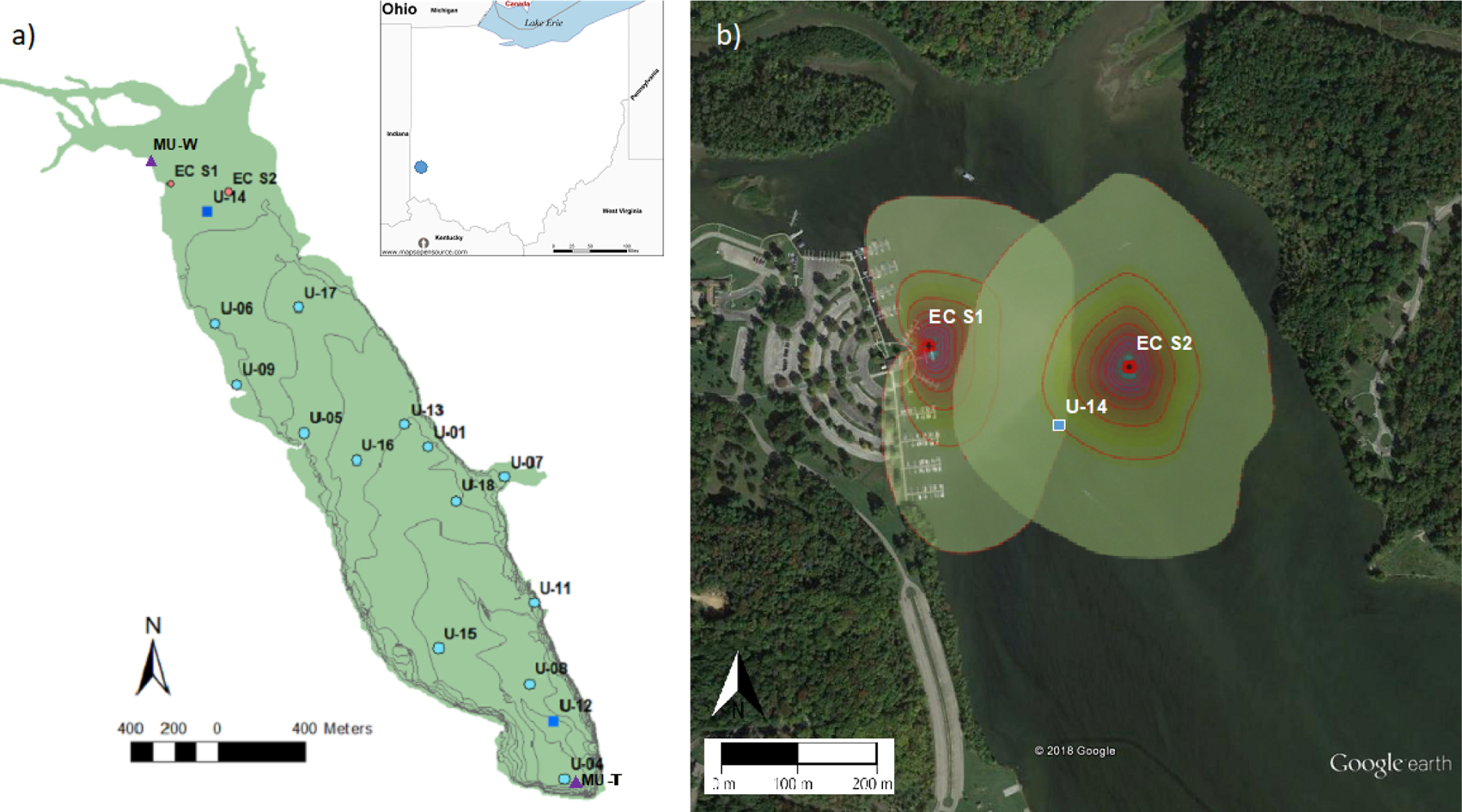
Map of Acton Lake **(a)**, showing the location of multiple monitoring methods: eddy covariance flux tower sites (red circles), active funnel traps and biweekly chamber measurements (dark blue squares), spatially extensive survey sites (light blue circles), and the weather station and thermistors operated by Miami University (purple triangles). The lake contour lines represent ~ 1 m depth increments. Inset image shows the location of Acton Lake in southwest Ohio. The Google Earth image **(b)** shows the 80 % cumulative footprint probability distribution at each eddy covariance flux tower site at 10 % intervals.

Acton Lake is a small hypereutrophic reservoir located in southwest Ohio (39.57° N, 84.74° W; 262 m a.s.l.; Fig. 1a). The dam was constructed in 1956, and the reservoir and surrounding state park have been managed by the Ohio Department of Natural Resources since 1957. The reservoir’s surface area is 2.4 km^2^, it has a maximum depth of ~8 m, and the area near the dam undergoes thermal stratification in the summer. Although Acton Lake is immediately surrounded by a forested state park, land use in its watershed is *>* 80 % agricultural, with the majority used for intensive row cropping (Renwick et al., 2018). We used four main methods to monitor CH_4_ fluxes ($F_{{\text{CH}}_{4}}$) from Acton Lake during 2017 and 2018: (1) the EC technique, (2) continuous ebullition monitoring with active funnel traps, (3) biweekly chamber measurements of diffusive emissions, and (4) spatially extensive surveys. The locations of the EC tower sites, active funnel trap and biweekly chamber measurement sites, and spatially extensive survey sites are depicted in Fig. 1a; the cumulative footprint probability distribution of the two flux tower sites is shown in Fig. 1b. The EC instrumentation was sited in the shallow region of Acton Lake due to logistical constraints related to both tower installation and boat traffic in the reservoir. How the methods were used in this study is summarized in Table S1. We used auxiliary meteorological and limnological measurements from stream gauging stations, a weather station, and thermistor string maintained by the Miami University (Renwick et al., 2018; Andersen et al., 2020), the locations of which are also shown in Fig. 1a.

### 2.2 Eddy covariance flux measurements

This site is registered as AmeriFlux site US-Act; information about the site and the flux data presented in this study are available online (https://ameriflux.lbl.gov/sites/siteinfo/US-Act, last access: 28 August 2021). The EC instrumentation consisted of an ultrasonic anemometer to measure three-dimensional wind speed and direction (Model 81000, R.M. Young Company, Traverse City, MI, USA) and open path infrared gas analyzers (IRGAs) for measuring the number density of CH_4_ (LI-7700), as well as CO_2_ and water vapor (LI-7500A, LI-COR Biosciences, Lincoln, NE, USA). The EC data streams were recorded at 10 Hz by a data logger (LI-7550, LI-COR Biosciences, Lincoln, NE, USA), which was also equipped with a temperature sensor and a pressure transducer. The EC system was deployed from a dock piling 20 m from the northwestern shore of Acton Lake from 1 February 2017 through 14 April 2018 (“EC S-1” in Fig. 1). The instruments were brought to the lab for calibration and maintenance on 15 April 2018, then redeployed on a tower installed into the reservoir sediment in the northeast corner of the reservoir on 5 May 2018 (“EC S-2” in Fig. 1). The system was shut down on 1 December 2018. Images of the EC system at each deployment location are included in the Supplement (Fig. S1). In addition to the EC setup, the flux tower was equipped with a net radiometer (NRLite2, Kipp and Zonen, Delft, The Netherlands), a cellular modem for remote communication (AirLink, Campbell Scientific, Logan, UT, USA), and a time-lapse camera (WCT-00125 Timelapse-Cam, Wingscapes, Calera, AL, USA). The time-lapse camera was used to determine periods of ice cover. The system was powered by solar panels and a battery bank regulated via a solar charge controller (SunSaver, Morningstar Corporation, Newtown, PA, USA). All components of the EC system were run on a 12 V system until relocation to the aquatic tower, when the EC setup (LI-7700, LI-7500A, and LI-7500 infrared gas analyzers; Model 81000 sonic anemometer) was retrofitted to run on 24 V.

The raw 10 Hz EC data were processed into 30 min fluxes using the software EddyPro v. 6.2 (LI-COR Biosciences, Lincoln, NE, USA). We used measurements of water depth from the Miami University weather station to determine instrument height above water surface on an hourly time step, integrated into the flux processing as a dynamic metadata file. Additional processing steps followed community standards and included filtering the 10 Hz CO_2_ measurements when CO_2_ signal strength was *<* 70, double coordinate rotation, block averaging, time lag compensation using covariance maximization, WPL density correction (Webb et al., 1980), and correction for high-pass and low-pass filtering effects (Moncrieff et al., 2004, 1997). The area contributing to the measured flux was characterized for both sites using the online two-dimensional flux-footprint prediction tool (Kljun et al., 2015). We used R for postprocessing, and the code is available on GitHub (https://github.com/USEPA/actonEC, last access: 28 August 2021). The 30 min fluxes were rejected when the period did not pass the tests for stationarity and developed turbulent conditions (quality control, QC, level 2 per the integrated scale of Foken et al., 2004). EC S-1 fluxes were further filtered for periods when winds were from the shore (between 195° and 33°); at EC S-2 we filtered for periods of low turbulence using a friction velocity (*u*_star_) threshold of 0.07 m s^−1^, based on the site-specific relationship between *u*_star_ and fluxes of CH_4_ and CO_2_ (Aubinet et al., 2012). We did not use *u*_star_ filtering at EC-S1 because the temporal coverage was insufficient to determine a *u*_star_ threshold. We define “acceptable” data or “acceptance rate” as those data meeting the EC QA/QC (quality assurance/quality control) requirements, while “data coverage” includes non-operability due to power or instrument failures.

The overall EC $F_{{\text{CH}}_{4}}$ data acceptance rate for the 2-year monitoring period (26 January 2017–13 November 2018) was 31.3 % (Fig. S2). In 2017, the data acceptance rate was lower, 23.4 %, due to power issues and the need to filter for wind direction at the near-shore EC S-1 site where the instrumentation was located for the whole year vs. 39.8 % in 2018 when the instrumentation was relocated in the spring to the mid-reservoir EC S-2 site. The data coverage for the period of monitoring from EC S-2 (May through November) was 52.8 %. Re-siting removed the need to filter periods based on wind direction and coincided with an improvement to the battery system that reduced incidences of power failure. At EC S-1, non-operability of the LI7700 due to power loss or other issues caused the majority of data rejection (40.4 % of total monitoring periods), followed by filtering for wind direction (28.1 %), and quality control filtering (7.8 %). At EC S-2, power loss caused the majority of gaps (36.3 %), followed by quality control filtering (16.6 %).

### 2.3 Active funnel trap ebullition measurements

The active funnel traps (AFTs) were based on the design of Varadharajan et al. (2010) and have been previously described by Beaulieu et al. (2018). Briefly, they consisted of a 0.3 m^2^ funnel attached to a rigid tubing gas collection chamber equipped with a differential pressure sensor to monitor accumulated gas volume on a 5 min time step. We modified the Varadharajan et al. (2010) design by incorporating siphons that auto-purge the collected bubble gas and refill the tubing volume with water. This modification keeps the AFTs from becoming filled with gas, allowing them to make useful measurements for longer periods of time. Trap gas samples were collected biweekly and analyzed via a gas chromatograph equipped with a flame ionization detector (Bruker 450 GC, USA) to determine the composition of the bubble gas. The active trap data reduction followed the method described in Varadharajan et al. (2010) and Varadharajan and Hemond (2012). Circuit calibration to determine the relationship between voltage and height was performed pre- and post-trap deployment in the 2017 field season and post-deployment in the 2018 field season. The volume of gas in the trap is calculated as follows:

(1) $${\text{AFT}}_{\text{vol}}=({\text{Circ}}_{\text{volt}}\times m+b)\times \pi \frac{{{\text{AFT}}_{\text{d}}}^{2}}{2},$$

where AFT_vol_ is the volume of gas in the funnel trap, Circ_volt_ is the voltage output from the differential pressure sensor, *m* and *b* are the sensor-specific laboratory calibration multiplier and offset coefficients, and AFT_d_ is the diameter of the funnel tubing. We used a 12-point moving average (60 min) to smooth the gas volumes and minimize noise. Periods with known issues were filtered out of the dataset (e.g., power issues, trap drift from target location, etc.), as were large negative fluxes that reflected siphon purges. Following Varadharajan and Hemond (2012), we calculated fluxes on multiple time-bin widths (30 min, 1, 2, 6, 12, 24, 48 h) but used the 2 h rolling time step for calculating the flux used in our final analysis:

(2) $$F_{{\text{CH}}_{4\text{eb}}}=\frac{{\text{AFT}}_{\text{vol}}[{\text{CH}}_{4}]}{(T_{\text{f}}-T_{\text{i}})A_{\text{F}}},$$

where AFT_vol_ is the volume of gas in the trap (m^3^), [CH_4_] is the CH_4_ concentration in the bubble gas (mg CH_4_ m^−3^), *T*_f_–*T*_i_ is the elapsed time (*s*), and *A*_F_ is the cross-sectional area of the funnel (m^2^). The AFT data reduction was performed in R, and the scripts are available online (https://github.com/USEPA/actonEC, last access: 7 September 2021).

The AFTs were deployed in late spring and retrieved in the fall each year. The shallow AFT (U-14) monitored ebullition from 9 May to 3 October in 2017 and from 6 June to 11 December in 2018. The deep AFT (U-12) monitored ebullition from 10 May to 30 October in 2017 and from 24 May to 9 November 2018.

### 2.4 Chamber diffusion measurements

Diffusive $F_{{\text{CH}}_{4}}$ was measured with a floating chamber biweekly at two sites during the field season. We used a rectangular, round-ended aluminum chamber with external polyvinyl chloride floats and a headspace fan, based on the CSIRO chamber described in Zhao et al. (2015). An ultraportable greenhouse gas analyzer (UGGA; PN: 915–0011, ABB, Los Gatos, CA) monitored the change in CH_4_ mixing ratio in the chamber headspace over the duration of the chamber deployment (*>* 1–5 min), measuring at 1Hz and recording an averaged measurement every 5 s. We monitored the real-time UGGA time series to prevent ebullitive emissions from overwhelming the diffusive emission measurements. If a spike in CH_4_ concentration was detected, we re-set the chamber. The floating chamber data reduction method has been described in detail in Beaulieu et al. (2016). Briefly, we used the following equation to calculate diffusive fluxes moles m^−2^ s^−1^):

(3) $$F_{{\text{gas}}_{\text{D}}}=\frac{{\text{dχ}}_{\text{gas}}}{\text{d}t}\left(\frac{V}{A}\right)\left(\frac{P}{RT}\right),$$

where d*χ*_gas_*/*d*t* is the rate of change in the mixing ratio of CH_4_ in the chamber headspace (ppm s^−1^), *V* is the chamber volume (m^3^), *A* is the chamber surface area (m^2^), *P* is the pressure in the chamber headspace, *R* is the universal gas constant, and *T* is the temperature in the chamber headspace. The rate of change d*χ*_gas_*/*d*t* for each chamber deployment was determined via fitting linear and nonlinear models to the dataset and using Akaike information criterion (AIC) to choose the more appropriate model. Only models with an *r*^2^ *>* 0.9 were retained. Data analysis and reduction was performed using *R*, and the scripts are available online (https://github.com/USEPA/actonEC, last access: 7 September 2021).

Biweekly chamber monitoring was conducted from 10 May to 11 December in 2017, and from 18 May to October to 13 December in 2018. Note that the chamber monitoring began earlier and ended later than the AFT monitoring each year due to technical issues with the AFTs.

### 2.5 Water measurements

Water temperature depth profiles were recorded continuously at two sites close to U-14 and U-12 (Fig. 1) using thermistors. At the shallow site (U-14) a string of seven thermistors (RBRsoloT, RBR Ltd., Ottawa, ON, Canada) were deployed at 0.1, 0.25, 0.5, 0.75, 1, and 1.5 m below the air–water interface and at the sediment–water interface. We used this temperature profile to characterize water column stability in the footprint of the EC flux measurements based on the Brunt–Väisälä buoyancy frequency using the R package rLakeAnalyzer (Winslow et al., 2019). The Brunt–Väisälä buoyancy frequency was used to indicate water column stability. It represents the frequency at which a parcel of fluid will oscillate when displaced vertically, a measure of resistance to mixing. A high oscillation frequency indicates strong resistance to mixing, whereas a low frequency indicates little resistance to mixing. At the deep site (U-12), sondes measuring temperature (ProODO, YSI Incorporated, Yellow Springs, OH, USA) were deployed at 0.1, 0.5, 1, 1.5, 2, 3, 4, 5, 6, 7, and 8 m below the air–water interface. Water temperature, specific conductivity, dissolved oxygen, pH, and chlorophyll *a* (chl *a*) were measured biweekly with a YSI multiparameter sonde at 0.1 and 1.5 m below surface at the shallow site (U-14) and 0.1, 1, 2, 3, 4, 5, 6, 7, and 8 m below surface at the deep site (U-12). Water samples for chlorophyll analysis were collected by Miami University near the reservoir inlet. Water samples were collected with an integrated tube sampler from the water surface to the euphotic zone depth. Chlorophyll samples were collected on 1.0 µm glass fiber filters and frozen at −20 °C in opaque containers until processed. They were extracted in 95 % ethanol for 24 h and analyzed with a TD-700 (Turner Designs, San Jose, CA, USA).

Dissolved gas surface and profile samples were collected biweekly from both U-12 and U-14 using the headspace equilibration method. We collected water samples at depths of 0.1, 2, 4, 6, and 7 m at U-12 and at 0.1, 0.75, and 1.3 m at U-14. Using a 140 mL plastic syringe with a two-way stopcock, we added 25 mL of ultra-high-purity helium to a syringe, then added 115 mL of sample water, and agitated all samples for 5 min. We then transferred the headspace gas to pre-evacuated 12 mL glass vials topped with a silicone-coated Teflon septum stacked on top of a chlorobutyl septum (Labco Ltd., UK). The headspace gas samples were analyzed using gas chromatography (see Sect. 2.3) to determine the CH_4_ composition, and the dissolved CH_4_ concentrations were calculated using measured headspace composition and the temperature-specific Bunsen solubility coefficients (Yamamoto et al., 1976). Full documentation of the calculations is available at the National Ecological Observatory Network’s GitHub repository (https://github.com/NEONScience/NEON-dissolved-gas, last access: 7 September 2021).

### 2.6 Whole-reservoir surveys

We conducted six surveys of Acton Lake over the summers of 2017 and 2018 to estimate whole-reservoir $F_{{\text{CH}}_{4}}$. The 15 sample collection sites (Fig. 1, light blue circles), were determined using a generalized random tessellation survey (GRTS) design (Stevens and Olsen 2004; Olsen et al., 2012), a probability design that has been shown to reduce uncertainty relative to other designs (Beaulieu et al., 2016). At each site, we measured CH_4_ diffusion, CH_4_ ebullition, and surface water quality parameters. Survey measurements of diffusive $F_{{\text{CH}}_{4}}$ were conducted with floating chambers in the same manner as described in Sect. 2.4. Survey measurements of ebullitive $F_{{\text{CH}}_{4}}$ were conducted with passive funnel traps (PFTs) deployed overnight (*>* 15 h). The PFTs are a simplified version of the AFTs described in Sect. 2.3: they consist of a 0.3 m^2^ funnel attached to a section of tubing for gas collection but do not have a pressure sensor or siphon. Upon retrieval, the total time of deployment and total volume of gas in the tubing were recorded, and three 25 mL samples of the gas were collected for gas composition analysis via a gas chromatograph (see Sect. 2.3). Ebullitive $F_{{\text{CH}}_{4}}$ from the PFTs was also calculated using Eq. (2) (Sect. 2.3), but the trap volume was determined by direct measurement of the collected gas, and *T*_f_–*T*_i_ is defined as the deployment period. Dissolved gas sample collection and depth profiles of water quality parameters were taken at one deep site (U-12) and one shallow site (U-14) during each whole-reservoir survey. The surveys were initiated on 10 July, 31 August, and 4 October 2017 and 10 July, 14 August, and 20 September 2018 and concluded the following day.

### 2.7 Gap filling and upscaling

We use the term “gap filling” to refer to our method to determine values for missing observations in our measurement time series, while “upscaling” refers to the best estimate of whole-reservoir $F_{{\text{CH}}_{4}}$. For this analysis, we separated the year into different seasons, categorizing November through March as “winter”, or the cold season, and May through September as “summer”, or the warm season. We refer to April and October as the “shoulder” season. The spring burst period is defined as 24 May through 4 June. For the EC time series, we developed an artificial neural network (ANN) to gap fill 30 min $F_{{\text{CH}}_{4}}$ using predictor variables with biophysical links to CH_4_ production and emission: sediment temperature (sedT), air temperature, latent heat flux (LE), sensible heat (*H* ), wind speed, *u*_star_ (friction velocity, a measure of turbulence), photosynthetically active radiation, overlying static pressure, and change in static pressure, in which static pressure is the sum of overlying atmospheric and hydrostatic pressure. We also included indicators for the tower location, hour of day, and day of year as drivers. Gaps in the sedT, air temperature, wind speed, wind direction, and static pressure time series were filled using observations from a nearby weather station. Gaps in LE, H, and *u*_star_ were gap filled using the mean diurnal course function from the R package REddyProc (Wutzler et al., 2019) on the 30 min time step. We used *k*-means clustering to assign 10 clusters before selecting the training, testing, and validation datasets. The cluster assignments allowed us to select subsets with probabilities proportional to the clusters, ensuring that the clusters were not over- or underrepresented as a result of the splits. We employed a selective ensemble approach to optimize the ANN model performance using the R package nnet (Venables and Ripley, 2020). Each ANN ensemble included models with 5–20 layers and 50 different starting weights, for a total of 800 model results. The top 100 models were selected based on the testing *R*^2^ results, and then the median CH_4_ value from the best 100 models was used as the predicted flux. To characterize both sampling and model uncertainty, we replicated this procedure with 20 resamplings of the data. For each half hourly $F_{{\text{CH}}_{4}}$ we calculated the median predicted value of the best 100 models in each of the 20 ensembles of 800 models (cf. Knox et al., 2016). Missing half hourly $F_{{\text{CH}}_{4}}$ values were gap filled using the median of the medians from the 20 ensembles. ANN modeling and gap filling was performed in R, and the scripts are available online (Barnett et al., 2021).

We gap filled short gaps in the AFT continuous datasets using linear interpolation and calculated annual emissions via summing the daily observations. We gap filled the biweekly chamber measurements of diffusive $F_{{\text{CH}}_{4}}$ via linear interpolation. For periods at the start and end of the monitoring seasons with chamber measurements but no AFT measurements, we used the typical ratio between diffusive and ebullitive $F_{{\text{CH}}_{4}}$ to estimate total $F_{{\text{CH}}_{4}}$ for the site. We gap filled the spatial survey measurements by interpolating between each of the three annual surveys. To estimate annual emission, we applied the $F_{{\text{CH}}_{4}}$ value determined by the first survey of the year to every day between 1 May and the first survey and the $F_{{\text{CH}}_{4}}$ value determined by the last survey of the year through 15 October. We assumed an $F_{{\text{CH}}_{4}}$ of zero between 15 October and 1 May for both the spatial survey dataset and the AFT plus chamber datasets.

To upscale to whole-reservoir $F_{{\text{CH}}_{4}}$, we used a hybrid approach, combining results from EC, the deep site (U-12) AFT, and the spatial surveys. We stratified Acton Lake into shallow (*<* 3 m) and deep (≥ 3 m) areas and used reservoir bathymetry to determine the surface area for the shallow and deep portions: 0.8 and 1.6 km^2^, respectively. The depth cut-off of 3 m roughly corresponds to the greatest depth of the EC footprint. We then used $F_{{\text{CH}}_{4}}$ measured by EC to characterize the shallow portion of the reservoir. For the deep portion, we calculated the ratio (reservoir ratio, or RR) between the measured $F_{{\text{CH}}_{4}}$ (ebullitive + diffusive) at the U-12 AFT (hereafter, deep AFT $F_{{\text{CH}}_{4}}$) and the mean of $F_{{\text{CH}}_{4}}$ measured at the other deep sites (U-01, U-04, U-05, U-08, U-11, U-12, U-13, U-15, U-16, U-17, and U-18; see Fig. 1). We calculated this RR for each of the six spatial survey dates. To characterize $F_{{\text{CH}}_{4}}$ in the deep portion of the reservoir, we applied the RR from the first survey to the deep AFT $F_{{\text{CH}}_{4}}$ continuous time series data collected before 10 July 2017 and likewise applied the RR from the last survey to the time series data collected after 20 September 2018. For the periods in between, we used linear interpolation to produce a daily RR and applied that to the deep AFT $F_{{\text{CH}}_{4}}$ continuous time series. We weighted the cumulative shallow and deep CH_4_ areal emissions by the shallow and deep fraction of the reservoir to determine the whole-reservoir CH_4_ emissions. We refer to this estimate of whole-reservoir emissions as the “hybrid” upscaled estimate.

### 2.8 Uncertainty analysis

We parameterized the uncertainty in the EC time series of $F_{{\text{CH}}_{4}}$ using three different measures: the random measurement error, the bias error of the gap-filled dataset, and the 95 % confidence intervals of the gap-filled dataset. The random measurement error is calculated from the variance of the covariance (Finkelstein and Sims, 2001) and reflects instrument noise, variation in footprint over a given 30 min flux integration period, and the stochastic nature of turbulence. As described in Jammet et al. (2017), the random error decreases with increasing dataset size and is negligible at the resolution of cumulative annual fluxes but can be substantial for individual flux measurements (Richardson et al., 2006; Moncrieff et al., 1996). The random error was calculated as part of the EddyPro processing, and we report the summary statistics in Sect. 3.2. Unlike random errors, systematic biases can accumulate to affect the cumulative seasonal or annual flux. Although the measurement bias cannot be quantified, we calculated the systematic bias in the annual fluxes due to gap filling following Moffat et al. (2007) and Jammet et al. (2017):

(4) $$\text{BE}=\frac{1}{N}\sum \left(p_{i}-o_{i}\right),$$

where *N* is the number of values in the validation time series, *p* is the values predicted by the ANN, and *o* is the observed values in the validation time series. The bias error was multiplied by the total number of gap-filled values to obtain the total annual bias. We calculated the 95% confidence interval of the gap-filled dataset using the distribution of the 20 ANN medians extracted from the 20 resamplings, which consider both sample and model uncertainty (Knox et al., 2016).

We used root-sum-squared error propagation of the error in AFT_vol_ and [CH_4_] to characterize the uncertainty in ebullitive $F_{{\text{CH}}_{4}}$ measured by the AFTs. Compared to error in AFT_vol_, the error contribution from other terms in Eq. (2) was negligible. As described in Varadharajan et al. (2010), we propagated the error in *m*, offset, and electronic noise through Eq. (1), adding a 2 mL dead volume error each time the AFTs flushed to account for gas that could be trapped in the fittings at the top of the collection chamber. Our mean slope and slope error were similar to those reported in the methods of the Varadharajan et al. (2010) paper (31 and 0.31, respectively, compared to 28 and 0.5); the mean (*V*_zero_) and standard deviation (*∆V*_zero_) of the offset terms we used were slightly larger: 0.51 and 0.071 V for the shallow site and 0.41 and 0.045 V for the deep site (compared to 0.15 and 0.015); our calculated electronic noise (*∆V*_out_) was smaller (0.4 mV vs. 3 mV in Varadharajan et al., 2010), so we defaulted to their value. The standard deviation between the multiple trap gas samples was used as the uncertainty in [CH_4_]. This term was generally small compared to the uncertainty due to AFTvol error. The cumulative errors were propagated by summing in quadrature.

The whole-reservoir surveys provide an estimate of $F_{{\text{CH}}_{4}}$ integrated across the entire reservoir surface area and a 95% confidence interval range (Beaulieu et al., 2016). Variance estimates calculated from GRTS incorporate spatial autocorrelation, if present, resulting in smaller uncertainty ranges than survey approaches that ignore spatial autocorrelation (Stevens and Olsen, 2003). The GRTS design and data reduction were executed in R using the spsurvey package (Kincaid et al., 2019). We propagated the cumulative uncertainties across 2017 and 2018 by taking the 95% confidence interval of each survey and summing them in quadrature.

The uncertainty in the hybrid approach to the upscaled cumulative whole-reservoir emissions was also determined by error propagation, combining the uncertainty in the deep AFT measurements, the spatial surveys, and the EC measurements.

### 2.9 Statistical and quantitative analysis

For these analyses, we used the non-gap-filled measurement time series. We quantified the relationship between sediment temperature (sedT) and $F_{{\text{CH}}_{4}}$ using *Q*_10_ and breakpoint analyses. The concept of an “ecological *Q*_10_” (DelSontro et al., 2016) follows from the physiological exponential relationship between metabolic processes and temperature. In contrast to physiological *Q*_10_ values, ecological *Q*_10_, hereafter “ecoQ10”, values are muddied by time lags and competing rate enhancers and inhibitors (e.g., that temperature affects both methanogens and methanotrophs; Segers, 1998; Duc et al., 2010; Lofton et al., 2014). While the physiological *Q*_10_ value for methanogenesis converges around 4 (Yvon-Durocher et al., 2014), ecoQ10 values for methane fluxes have been reported to range from 1 to 35 (e.g., DelSontro et al., 2016; Wik et al., 2014; Duc et al., 2010). We calculated the ecological *Q*_10_ (DelSontro et al., 2016) using the following equation:

(5) $$\text{ecoQ}10=10^{10b},$$

where *b* is the slope of the regression between temperature and $F_{{\text{CH}}_{4}}$.

We also used a two-dimensional Kolmogorov–Smirnov test (2DKS; Garvey et al, 1998) to quantify the temperature breakpoint distinguishing winter conditions when $F_{{\text{CH}}_{4}}$ is near zero and unrelated to temperature from warm weather conditions when $F_{{\text{CH}}_{4}}$ is elevated and positively correlated with temperature. The 2DKS test is a non-parametric statistic that uses measures of disagreement to define the largest difference between cumulative distribution functions, that is, a threshold or breakpoint (Lopes et al., 2008). We applied the 2DKS test to each of the continuous $F_{{\text{CH}}_{4}}$ monitoring datasets: EC, shallow AFT, and deep AFT, each for 2017 and 2018 for a total of six 2DKS tests.

We looked at diurnal patterns on monthly and daily timescales. For the monthly timescales we binned 30 min periods and took the median. For daily timescales we adapted the methods used by Podgrajsek et al. (2014) to quantify “strong” diurnal patterns. For 24 h periods with at least eight nighttime and eight daytime non-gap-filled 30 min flux measurements, we compared the median of daytime $F_{{\text{CH}}_{4}}$ to nighttime $F_{{\text{CH}}_{4}}$. The period was defined as having a strong diurnal pattern both if the difference between daytime vs. nighttime $F_{{\text{CH}}_{4}}$ median was *>* 50 % and if the contiguous points in the 30 min time series were smooth, i.e., more similar than points separated in time. We determined smoothness using visual inspection.

We compared the cumulative $F_{{\text{CH}}_{4}}$ measured from Acton Lake during each year of this study to output from the size-productivity model (DelSontro et al., 2018a). This model relates total CH_4_ emissions to chl *a* levels per the following equation:

(6) $$\log\;10\;(\text{total}\;{\text{CH}}_{4}+1)={\text{C}}_{1}\times \log\;10(\text{chl}\;a)+{\text{C}}_{2},$$

where the coefficients C_1_ and C_2_ are equal to 0.778 ± 0.118 and 0.940 ± 0.122, respectively. Although the equation is unitless, it relates total CH_4_ in units of milligrams C per square meter per day (mg C m^−2^ d^−1^) to chl *a* in units of micrograms per liter (µg L^−1^).

## 3 Results

### 3.1 Temporal patterns in $F_{\text{C}{\text{H}}_{\text{4}}}$

**Figure 2.**
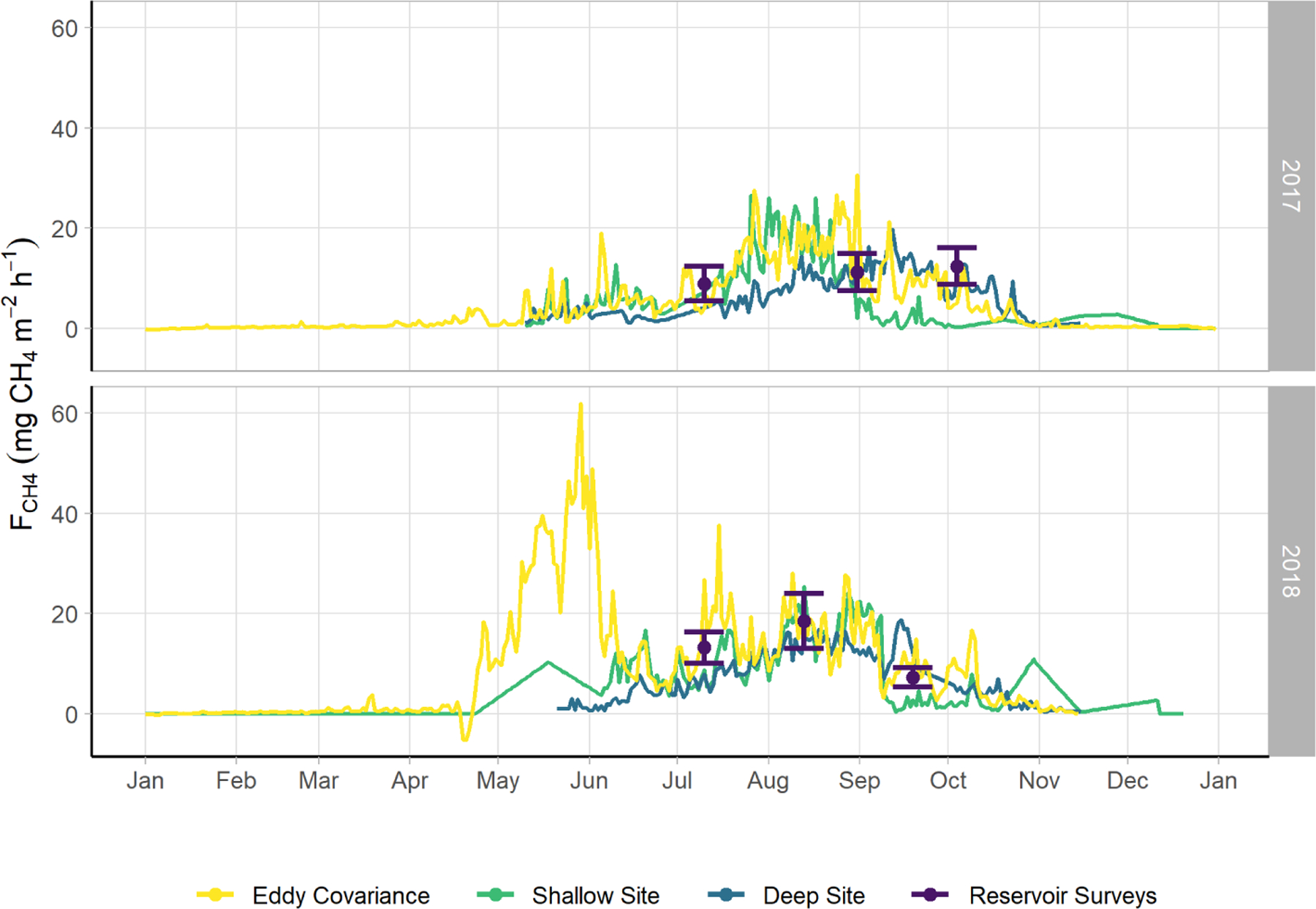
Time series of $F_{{\text{CH}}_{4}}$ monitored via multiple methods: eddy covariance (violet), the sum of the shallow AFT and interpolated chamber measurements (blue, site U-14), the sum of the deep AFT and interpolated chamber measurements (green, site U-12), and via the spatially integrated lake-wide surveys (yellow). The error bars for the lake surveys indicate the 95% confidence interval of the mean. Error margins for the other measurements are omitted for figure legibility. The spring burst period was 24 May–4 June 2018.

We observed a consistent pattern of elevated $F_{{\text{CH}}_{4}}$ during the warm season across all measurement methods (Fig. 2). In both monitoring years, the majority of cumulative total CH_4_ emissions (*>* 85 %) occurred in the 5 months between 1 May and 30 September, when air and sediment temperatures were warmer (Fig. 4a), and latent heat fluxes were elevated (Fig. 4b). We observed larger-magnitude CH_4_ emissions in 2018 relative to 2017 at Acton Lake across each observation type except for the deep site (Table 1). The EC and spatial survey results indicated similar warm-season mean fluxes in 2017: 9.73 ± 0.67 and 9.98 ± 6.2 Mg CH_4_ m^−2^ h^−1^. Results from both methods indicated larger-magnitude mean $F_{{\text{CH}}_{4}}$ in 2018: 17.5 ± 0.38 Mg CH_4_ m^−2^ h^−1^ per the EC system and 13.0 ± 6.6 Mg CH_4_ m^−2^ h^−1^ per the spatial surveys (Table 1). Both the shallow site results also indicated elevated $F_{{\text{CH}}_{4}}$ in 2018 relative to 2017, while the deep site results were effectively the same (Table 1). The lower-magnitude mean $F_{{\text{CH}}_{4}}$ measured at the shallow site compared to the mean $F_{{\text{CH}}_{4}}$ measured by EC is likely due to the under-representation of hot spots (Wik et al., 2016). The wintertime $F_{{\text{CH}}_{4}}$ measured by EC indicates that during the winter months $F_{{\text{CH}}_{4}}$ dropped by more than an order of magnitude to a baseline close to zero: between 1 November and 1 April $F_{{\text{CH}}_{4}}$ was 0.60 ± 0.69 Mg CH_4_ m^−2^ h^−1^. The surface of Acton Lake was frozen for several periods during the 2017–2018 winter: 27 December 2017–10 January; 13–21 January; and 5–15 February 2018, during which $F_{{\text{CH}}_{4}}$ was 0.08 ± 0.46 Mg CH_4_ m^−2^ h^−1^.

**Figure 3.**
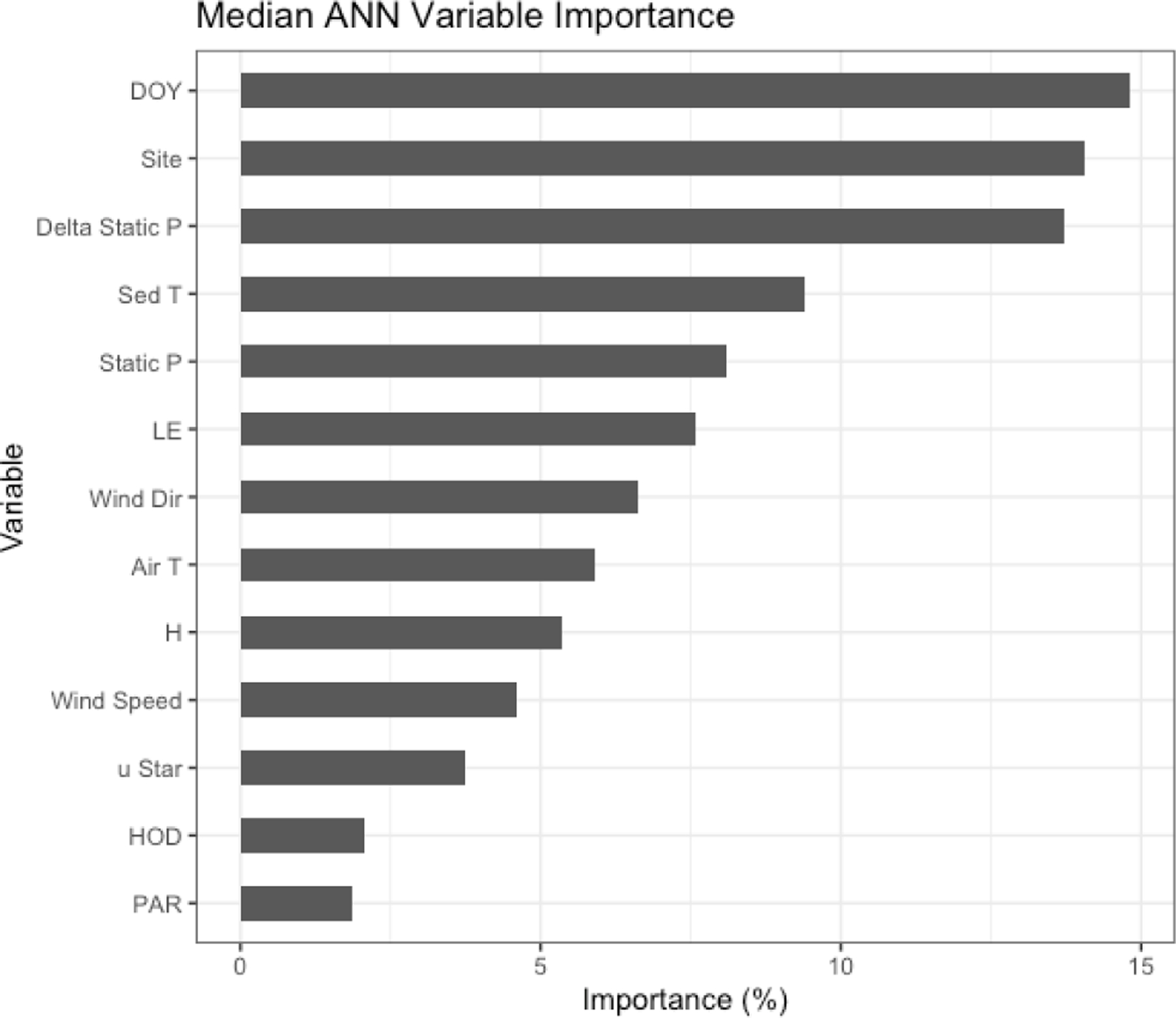
Median variable importance ranking for the drivers of the artificial neural network gap-filling model in terms of percent importance to the predictive power of the model. This ranking is based both on intra-model variability (i.e., the effect of model architecture and random seed selection) and on intermodel variability (i.e., the effect of data selection for the training, testing, and validation datasets). DOY = day of year, Delta Static *P* is change in overlying static pressure, sedT is sediment temperature, LE is latent heat flux, Static *P* is static pressure, Wind Dir is wind direction, *H* is sensible heat flux, *u*_Star_ is friction velocity, PAR is photosynthetically active radiation, and HOD is hour of day.

The non-gap-filled, quality-filtered 30 min $F_{{\text{CH}}_{4}}$ measurements had a mean random error (±SD) of 1.3 ± 1.9 and 1.8 ± 1.7 Mg CH_4_ m^−2^ h^−1^ in 2017 and 2018, respectively, or 15.5 % and 13.7 % of the mean annual fluxes. The fractional errors were larger in the winter months when $F_{{\text{CH}}_{4}}$ was small (mean winter random error: 23 %) and smaller during the warmer months when $F_{{\text{CH}}_{4}}$ was larger (mean summer random error: 15 %). Both the magnitudes and patterns in the random errors are similar to those observed by Jammet et al. (2017) in a subarctic aquatic ecosystem. Similarly, we found gap filling our $F_{{\text{CH}}_{4}}$ time series with ANN worked well with a few exceptions. The median *R*^2^ value for the 20 extractions was 0.79, and the cumulative bias error was minimal: the 20 ANN extractions yielded a median bias of 0.25 (range of −3.7 to 3.5) g CH_4_ m^−2^ or up to 3.3 % of cumulative emissions over the 2-year monitoring period. The ANN establishes nonlinear predictive power to each of the driver inputs, defined as a “variable importance factor” (VIF) in terms of a percent importance to the predictive power of the model. The median VIFs from the 20 ANN extractions are plotted in Fig. 3; a consistently high ranking across runs indicates a strong relationship with $F_{{\text{CH}}_{4}}$. The biophysical drivers with the highest variable importance were static pressure (the sum of water pressure and air pressure), change in static pressure, and sediment temperature.

The most substantial difference between the two monitoring years is the period of elevated emissions in late May to early June observed by the EC monitoring in 2018 but not 2017 (hereafter “spring burst”). We define the spring burst as the period from 24 May through 4 June, in which the daily average $F_{{\text{CH}}_{4}}$ observed by EC was ≥ 25 Mg CH_4_ m^−2^ h^−1^. Maximum $F_{{\text{CH}}_{4}}$ of 62.0 Mg CH_4_ m^−2^ h^−1^ occurred on 29 May 2018. While the 2017 EC monitoring does indicate a small burst in $F_{{\text{CH}}_{4}}$ of 20.4 Mg CH_4_ m^−2^ h^−1^ on 5 June, overall $F_{{\text{CH}}_{4}}$ was much smaller: mean $F_{{\text{CH}}_{4}}$ for 24 May–4 June 2017 was 3.6 ± 1.8 Mg CH_4_ m^−2^ h^−1^. Although the AFT at the shallow site was not operational during the spring burst, diffusive $F_{{\text{CH}}_{4}}$ measurements indicate that $F_{{\text{CH}}_{4}}$ was elevated at that site compared to the deep site. Although none of the spatial surveys coincided with the spring burst period, the deep site monitoring indicates that the spring burst did not extend to the deeper parts of the reservoir. The cumulative CH_4_ emission over the 2018 12 d spring burst period was 10.8 g CH_4_ m^−2^ which is 15 % of the cumulative annual emissions measured by EC in 2018 (Table 1) and which accounts for 59 % of the difference in the EC cumulative annual emissions between 2017 and 2018.

**Figure 4.**
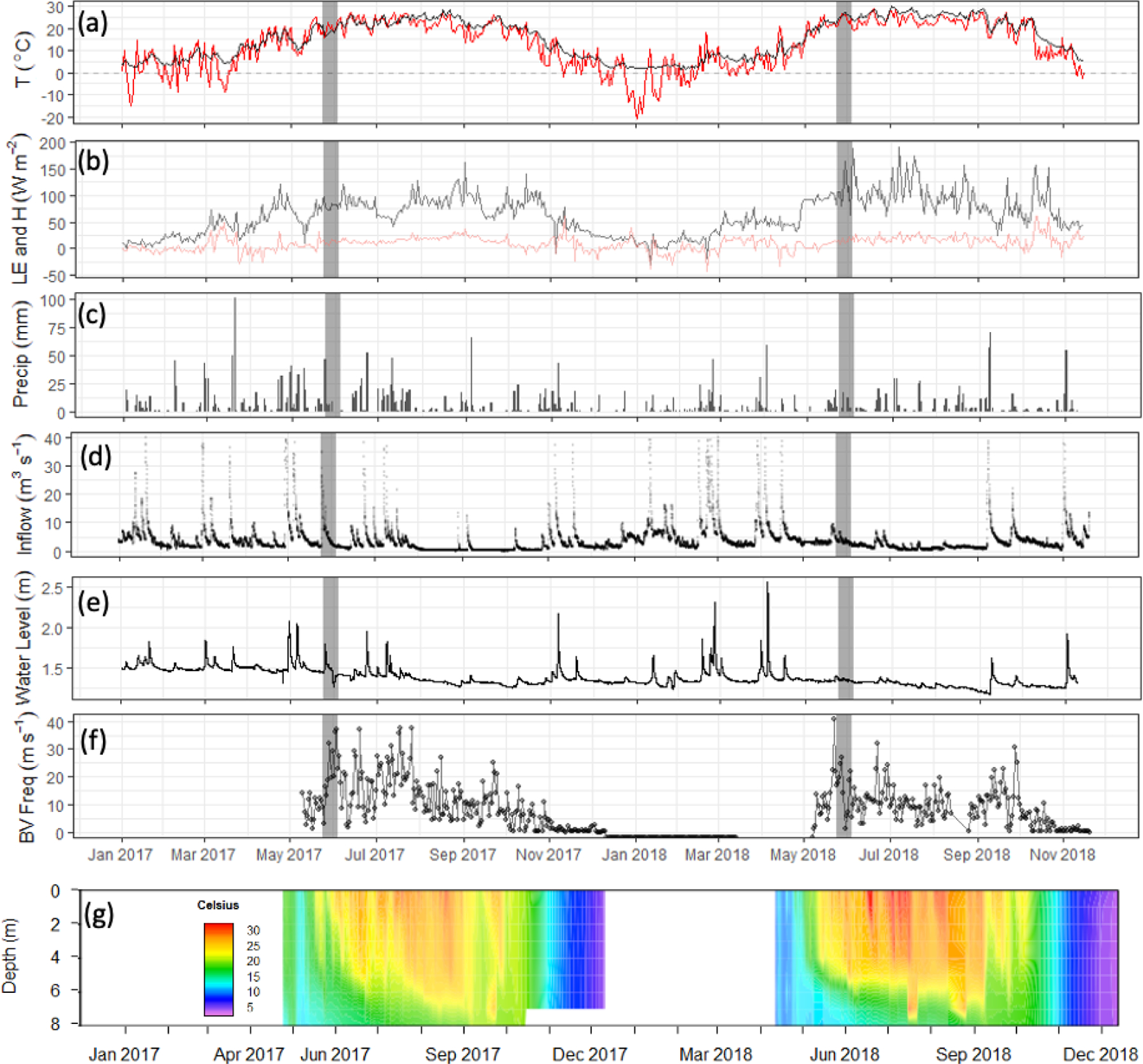
Meteorological and limnological conditions over the study period: **(a)** daily mean of air (red) and sediment (black) temperature; **(b)** daily mean latent and sensible heat fluxes (LE: black; *H* : red); **(c)** daily cumulative precipitation (mm); **(d)** stream inflow (m^3^ s^−1^); **(e)** water depth in the footprint of the flux tower (m); **(f)** Brunt–Väisälä frequency, a measure of water column mixing potential (s^−1^); and **(g)** the water temperature profile at the deep site (U-12). Grey bars indicate the time frame of the 2018 spring burst of CH_4_ emissions.

The differences between the 2017 and 2018 monitoring years continue past the early summer (Figs. 2, 4). During 2017, $F_{{\text{CH}}_{4}}$ increased to a maximum in late summer, and then declined back to the winter baseline. Maximum emissions at the deep site in 2017 lagged and were dampened compared to the shallow site. In contrast, the 2018 summer and fall in the shallow portion of the reservoir (EC and shallow site) were characterized by episodic emission pulses and declines before tapering down to the winter baseline. The deep site emissions were in phase with the shallow site but did not have the same pulses. There was a late season pulse at the deep site in 2018 that coincided with reservoir turnover (Fig. 4g) and a drop in dissolved CH_4_ below the thermocline at the deep site (Fig. S3).

We used the EC measurements of $F_{{\text{CH}}_{4}}$ to look for diurnal patterns in emissions. We found that Acton Lake did not have a clear overarching diurnal pattern when aggregated over monthly timescales, (Fig. S4), but out of the 168 d with adequate data coverage for diurnal analysis, 18.5 % (31 d) displayed strong diurnal patterns: 16 with elevated daytime emissions and 15 with elevated nocturnal emissions. Very few of these strong diurnal pattern days were contiguous: there were only four instances of strong diurnal patterns persisting for 2 or more consecutive days. The periods with strong diurnal patters when $F_{{\text{CH}}_{4}}$ peaked during the day were correlated with latent heat flux (Figs. S5, S6), while periods when $F_{{\text{CH}}_{4}}$ peaked at night were correlated with air pressure (Figs. S5, S6). While we looked for evidence of synoptic patterns in $F_{{\text{CH}}_{4}}$ due to changes in overlying pressure from frontal systems (cf. Liu et al., 2016) and due to underwater turbulence (Fig. S7), we did not see evidence of impact on $F_{{\text{CH}}_{4}}$ from these drivers during the study period.

### 3.2 Cumulative $F_{\text{C}{\text{H}}_{\text{4}}}$

**Figure 5.**
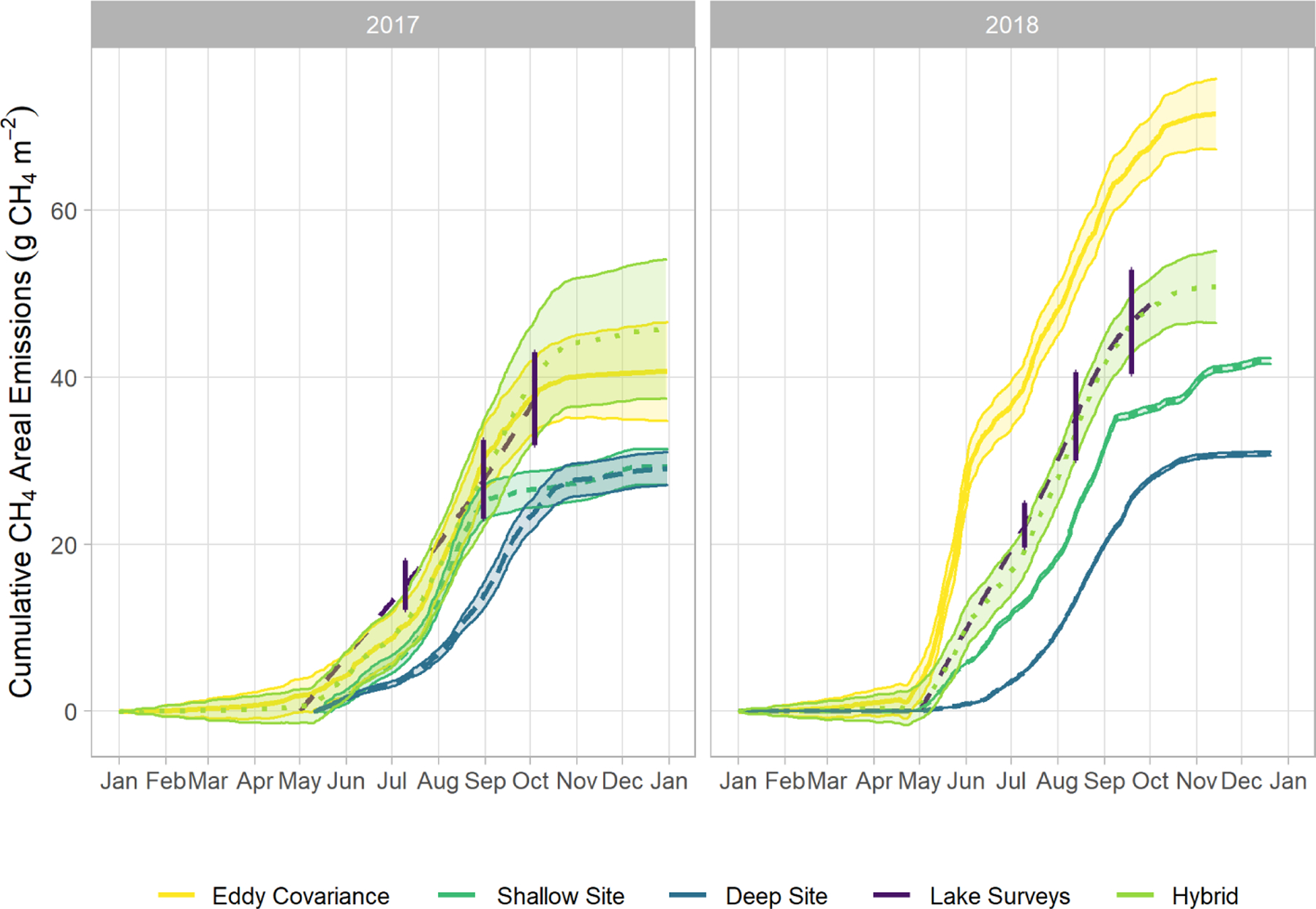
Cumulative areal emissions in 2017 and 2018 from EC, sum of AFT and chamber, spatial survey monitoring, and hybrid upscaling results (g CH4 m^−2^). Vertical lines intersecting the lake survey trace represent the 95% confidence interval of the lake-wide $F_{{\text{CH}}_{4}}$ estimate.

There are notable differences in the cumulative annual areal emissions across methods and years (Table 1, Fig. 5). The impact of the spring burst is evident in the interannual difference between the EC cumulative emissions, which were 40.7 ± 5.88 and 71.4 ± 4.2 g CH_4_ m^−2^ in 2017 and 2018, respectively. The cumulative areal emission measured by EC from 1 October 2017 through 1 May 2018 was 6.66 ± 3.1 g CH_4_ m^−2^, on the same order as the uncertainty range in the annual values. As follows from the patterns in the mean fluxes discussed above, the results from the spatial surveys and the shallow trap also indicate elevated cumulative annual emissions in 2018 compared to 2017, while the results from the deep site indicate similar emissions over both years. The implications of the spring burst for whole-reservoir upscaled total annual CH_4_ emissions is discussed below, but the best estimate of reservoir-wide cumulative annual areal emissions from the hybrid approach yields 45.6 ± 8.3 and 51.4 ± 4.3 g CH_4_ m^−2^ for 2017 and 2018, respectively (Fig. 5). Scaling up to the 2.4 km^2^ area of Acton Lake, the hybrid approach indicates that this reservoir was a source of 109 ± 14 and 122 ± 10 Mg CH_4_ to the atmosphere in 2017 and 2018, respectively.

### 3.3 Spatial patterns in $F_{{\text{CH}}_{4}}$

**Figure 6.**
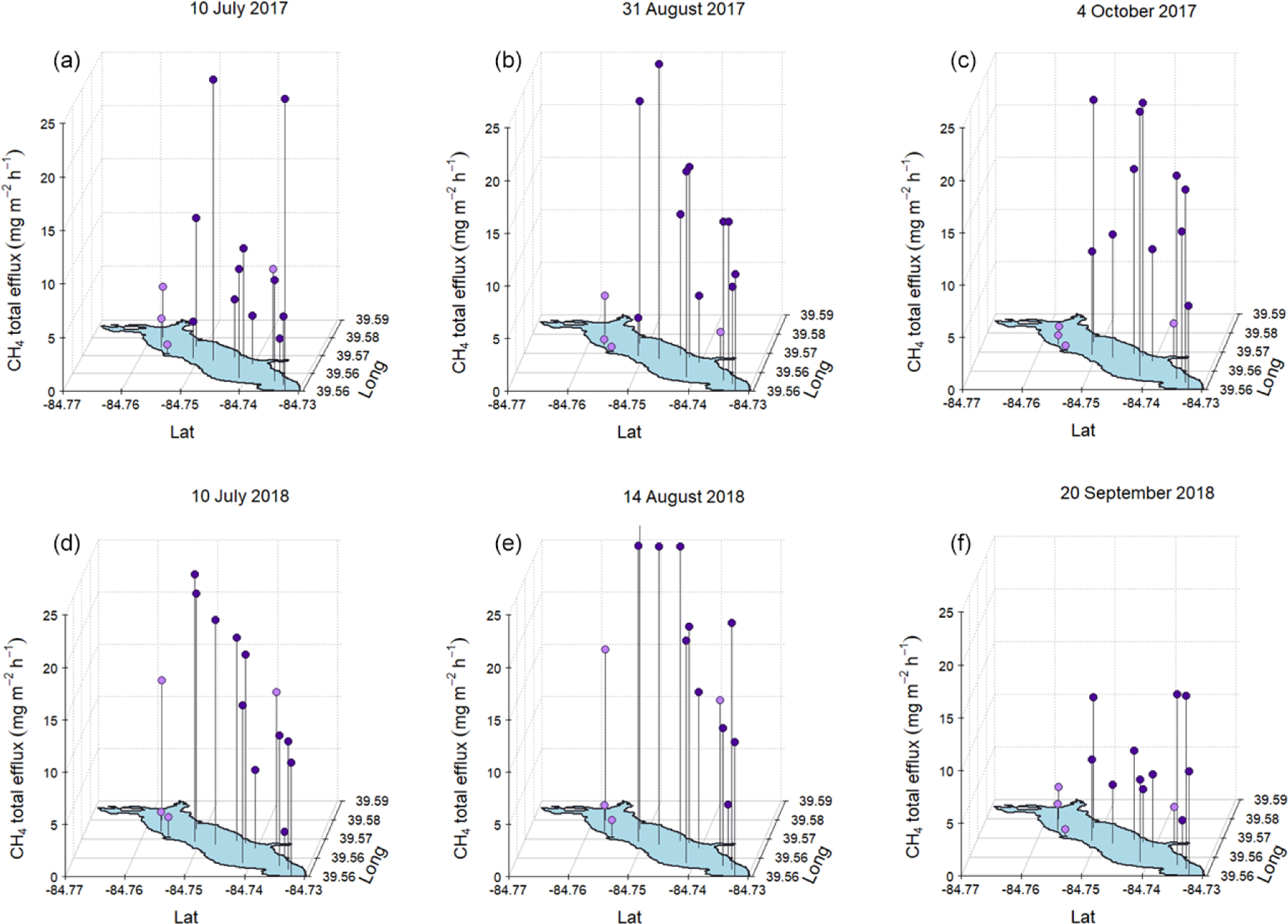
Total (ebullitive + diffusive) $F_{{\text{CH}}_{4}}$ measured during mid-summer, late-summer, and fall spatial surveys at Acton Lake during 2017 **(a, b, c)** and 2018 **(d, e, f)**. Dots indicate magnitude of $F_{{\text{CH}}_{4}}$ per the *z*-axis scale, and vertical black lines connect red dots to their corresponding sampling location. Dot color indicates whether a sampling site is in the shallow (*<* 3 m, lavender) or deep (*>* 3 m, royal purple) area of the reservoir.

The results from the six spatial surveys indicate an inconsistent spatial pattern in $F_{{\text{CH}}_{4}}$ that differs from previous findings on CH_4_ emissions from temperate, eutrophic reservoirs which have shown that the river–reservoir transition zone near the tributary inlets tends to be a hot spot for emissions compared to the lacustrine zone (Beaulieu et al., 2014, 2016; DelSontro et al., 2011; Tuser et al., 2017). The survey results from Acton Lake indicate relatively similar rates of $F_{{\text{CH}}_{4}}$ across most of the reservoir surface area (Fig. 6) and a weak but significant (*n =* 90, *R*^2^ = 0.1, *p <* 0.005) positive relationship between ebullition and reservoir depth (Fig. S8).

At the whole-reservoir scale, ebullition was a dominant emission pathway for CH_4_ relative to diffusion, accounting for 82 %–94 % of total $F_{{\text{CH}}_{4}}$. However, at certain sites diffusive $F_{{\text{CH}}_{4}}$ contributed a larger proportion of the total flux (Fig. S9). The four sites with mean ebullitive to total $F_{{\text{CH}}_{4}}$ ratios less than 0.8 are also the four shallowest sites (see Fig. 1): U-09, U-14, U-07, and U-06, with mean observed depths of 1, 1.3, 1.5, and 2 m respectively. This pattern from the spatial surveys is also reflected in the results from the more frequent measurements made at the shallow and deep site: ebullition accounted for 58 % of the total $F_{{\text{CH}}_{4}}$ at the shallow site in both 2017 and 2018, while ebullition accounted for 86 % and 88 % of total $F_{{\text{CH}}_{4}}$ at the deep site in 2017 and 2018, respectively. Emission behavior at sites U-09 and U-06 was substantially different than at other sites: these two sites had consistently low $F_{{\text{CH}}_{4}}$ and tended to have higher rates of CH_4_ diffusion than ebullition. Much of this behavior is likely explained by the proximity of these sites to Acton Lake’s swimming beach, which has a sandy substrate that likely inhibits methanogenesis at these sites. These sites were included as part of the random GRTS sampling design.

## 4 Discussion

### 4.1 Comparison with other systems and methods

The hybrid upscaling approach we used in this study leverages the best available information from our measurements to characterize both the spatial and temporal variability of Acton Lake: EC monitoring for the shallow portion of the reservoir and the continuous deep site monitoring scaled by the spatial survey site measurements for the deep portion of the reservoir. If we used the EC monitoring results alone to upscale to whole-reservoir emissions, that would assume the spring burst pattern affected the whole reservoir (Fig. 5). However, we know the spring burst did not affect the deep site (Fig. 2). Thus, a key uncertainty around this upscaling method is estimating what portion of the reservoir was affected by the spring burst of emissions in 2018. The cumulative $F_{{\text{CH}}_{4}}$ measured by EC was 77 % greater in 2018 than 2017, compared to a difference of only 11 % per the hybrid approach. Adding one or more AFT sites along the depth gradient of the reservoir would be one way to decrease uncertainty in the extent of the spring burst and improve confidence in upscaled $F_{{\text{CH}}_{4}}$ estimates.

Comparing cumulative annual areal emissions from the hybrid upscaling approach (45.6 ± 8.3 and 51.4 ± 4.3 g CH_4_ m^−2^ for 2017 and 2018, respectively) to other reservoir CH_4_ emission rates reported in the literature is not straightforward due to differences in monitoring methods and temporal coverage. One important reason earlier studies of reservoir $F_{{\text{CH}}_{4}}$ may be biased low is that they only measured CH_4_ diffusion: Deemer at al. (2016) found that the mean $F_{{\text{CH}}_{4}}$ reported in studies measuring ebullition and diffusion was over double that of diffusion-only $F_{{\text{CH}}_{4}}$ studies. Another potentially important source of bias is temporal coverage. Most studies that report $F_{{\text{CH}}_{4}}$ from inland waters monitor during the warm season, with less than 6 months of measurements (cf. Deemer et al., 2016; DelSontro et al., 2018a; Bastviken et al., 2011), and the mean $F_{{\text{CH}}_{4}}$ value is then extrapolated to annual total emissions. However, we measured very low (on the same order as the warm-season uncertainty) wintertime $F_{{\text{CH}}_{4}}$ in this study. On the other hand, the spring burst phenomenon we observed demonstrates the importance of continuous monitoring of midlatitude eutrophic reservoirs during the full warm season to capture hot moments of $F_{{\text{CH}}_{4}}$. A related consideration is a method’s ability to capture spatial and temporal variability in $F_{{\text{CH}}_{4}}$ during the study period. Deemer et al. (2016) noted that studies using the eddy covariance method reported substantially higher values of *F*_CH_: ∼ 92.5 g CH_4_ m^−2^ yr^−1^ (Deshmukh et al., 2014) and ∼ 160 g CH_4_ m^−2^ yr^−1^ (Eugster et al., 2011), which are on the same order as the Acton Lake cumulative annual emissions (Table 1). The two open-water sites included in the CH_4_ EC meta-analysis by Knox et al. (2019) were natural lakes in temperate regions with cumulative annual emissions of ~ 15 g CH_4_ m^−2^ yr^−1^. This difference in $F_{{\text{CH}}_{4}}$ speaks to the need for building a representative dataset across both methods and ecoregions.

Nevertheless, Acton Lake’s annual $F_{{\text{CH}}_{4}}$ is relatively high compared to other reservoirs. It falls in the fourth quintile (*>* 60 %) of the reservoir emission rates that included ebullition reported in Deemer et al. (2016); the warm season $F_{{\text{CH}}_{4}}$ falls in the upper quintile (*>* 80 %) of those reservoirs. The warm season $F_{{\text{CH}}_{4}}$ also falls into the upper quartile (*>* 75 %) of the 32 temperate reservoirs surveyed by Beaulieu et al. (2020). This result strengthens the finding that midlatitude, eutrophic reservoirs in the midwestern USA can support high CH_4_ emission rates (cf. Beaulieu at al., 2014, 2016) than would be predicted by age and latitude alone (Barros et al., 2012). The high annual $F_{{\text{CH}}_{4}}$ also supports the emerging body of knowledge around the importance of reservoir productivity as a key indicator for $F_{{\text{CH}}_{4}}$ (cf. Deemer et al., 2016; West et al., 2012; DelSontro et al., 2018b).

### 4.2 Implications for upscaling

The key question in upscaling any set of measurements to characterize an ecosystem is “what is representative of reality?”. This study leveraged a combination of continuous and spatially extensive monitoring methods to investigate the spatial and temporal variability in a reservoir. The results from the six spatial surveys indicate an inconsistent spatial pattern in $F_{{\text{CH}}_{4}}$ that differs from previous findings on CH_4_ emissions from temperate, eutrophic reservoirs which have shown that the river–reservoir transition zone near the tributary inlets tends to be a hot spot for emissions compared to the lacustrine zone (Beaulieu et al., 2014, 2016; DelSontro et al., 2011; Tuser et al., 2017). The spring burst of elevated emissions that we observed in 2018 but not 2017, and in the shallow portion of the reservoir but not at the deep site, is the largest contributor to the spatial and temporal variability in this study. In this section we will analyze the spring burst and factors that could have contributed to it. Other patterns in intra-reservoir spatial and temporal variability linked to sediment temperature and other biophysical drivers are also discussed.

#### 4.2.1 Spring burst

**Figure 7.**
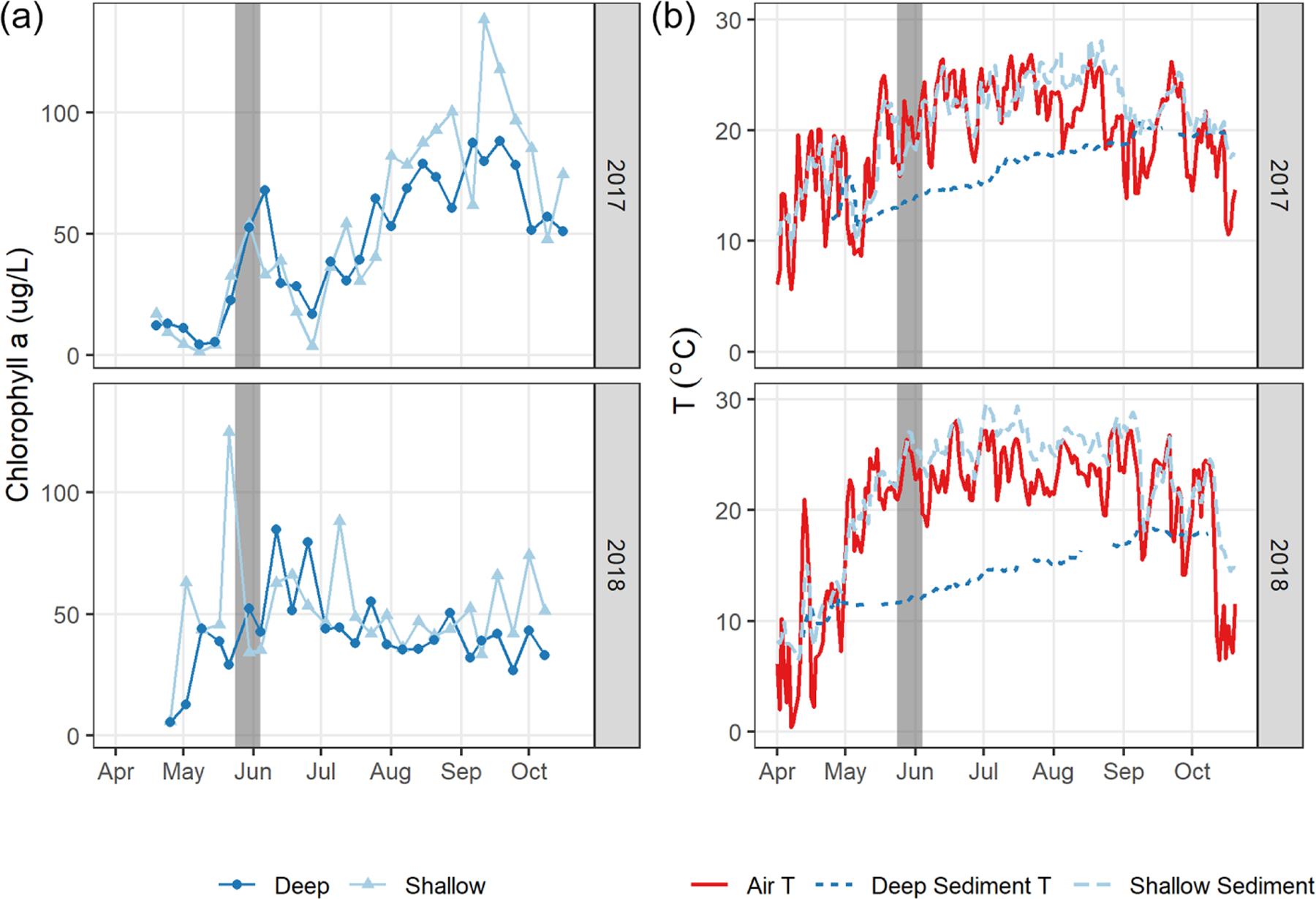
Daily air and sediment temperature (**a**, left) and chlorophyll *a* (an indicator for algal biomass, **b**, right) in 2017 and 2018. The grey bar indicates the spring burst period of elevated $F_{{\text{CH}}_{4}}$ in 2018, likely supported by elevated sediment temperature and algal biomass levels that year.

Differences in phytoplankton populations and sediment temperature, partially driven by precipitation differences, provide insight into why the spring burst of emissions occurred (1) in 2018 but not 2017 and (2) in the littoral area of the reservoir but not the deeper areas. Chlorophyll *a* (chl *a*) levels measured a few days before the spring burst period show elevated levels in the shallow portion of the reservoir in 2018 compared to 2017, while levels near the outflow were similar between the two years (Fig. 7a). This increase in chl *a* levels coincided with an increase in shallow sedT to 27 °C, (Fig. 7b). These differences in chl *a* and sedT near the inflow can be tied to differences in precipitation between the two years: spring of 2017 was relatively wet, with 31.0 cm of rainfall and 20.9 × 10^6^ m^3^ of stream inflow in May (Fig. 4c, d) which drove substantial fluctuations in reservoir water levels (Fig. 4e). These rain events also led to a decrease in sedT from 22.5 to 18 °C prior to the onset of the spring burst timeframe (Fig. 7b) due to the inflow of cooler stream water and the cooling of ambient air temperature. In contrast, May of 2018 was relatively dry, with 12.3 cm of rain, 9.45 × 10^6^ m^3^ of stream inflow (Fig. 4c, d), and stable reservoir water levels (Fig. 4e). The phytoplankton bloom in the shallow portion of the reservoir leading up to the spring burst period was likely catalyzed by the conducive water temperature, turbidity, and water level stability. Elevated levels of dissolved ammonium (NH_4_), total phosphorous (TP), soluble reactive phosphorus (SRP), and particulate organic carbon (POC) near the inflow during the 2018 spring burst support our understanding that the conditions in the littoral area in 2018 were different than those in 2017 and that this interannual difference did not occur in the deep portion of the reservoir (Table 2).

There are at least two established mechanistic connections between phytoplankton blooms and enhanced CH_4_ production and emission, and either or both could have driven the spring burst observed in this study. One mechanistic connection between autochthonous organic carbon (autoOC, i.e., phytoplankton-derived) and $F_{{\text{CH}}_{4}}$ is the stimulation of methanogenesis from the input of this labile C source as the phytoplankton die and settle on the sediment. Several lab studies have demonstrated that the addition of autoOC can lead to enhanced CH_4_ production rates (Schwartz et al., 2008; West et al., 2012, 2015; Grasset et al., 2018). A recent study using in situ measurements found that heat-wave-induced cyanobacterial blooms and subsequent input of autoOC to the sediment could lead to pulses of CH_4_ emissions up to an order of magnitude larger than baseline levels (Bartosiewicz et al., 2021). The 2018 crash in phytoplankton that coincided with the spring burst (as indicated by chl *a* measurements; Fig. 7a) evidences a large input of autoOC to the sediment during the spring burst. A second possible mechanistic connection is production of CH_4_ by phytoplankton in the oxic surface water. A recent study by Hartmann et al. (2020) combined in situ measurements of phytoplankton communities, CH_4_, and CH_4_ isotopes with lab incubations and demonstrated that all major phytoplankton classes could produce CH_4_ under oxic conditions. Phytoplankton CH_4_ production in the surface mixed layer supersaturates the upper water column with CH_4_ and leads to enhanced diffusive emissions, and phytoplankton biomass has been found to be the primary driver of diffusive $F_{{\text{CH}}_{4}}$ in some reservoir systems (McClure et al., 2020). Strong diurnal patterns in $F_{{\text{CH}}_{4}}$ surrounding the spring burst correlated with latent heat flux (LE), an indicator of warm, windy, convective conditions of enhanced air–water gas exchange (Figs. S5, S6). This suggests that during the spring burst the surface waters were supersaturated with CH_4_ and diffusive emissions were the dominant pathway during that time. Including measures of phytoplankton CH_4_ production in the surface mixed layer in future studies would be helpful in differentiating which production pathway led to elevated dissolved CH_4_.

The difference in hydrologic regimes and subsequent availability of autoOC vs. allochthonous OC (alloOC, i.e., particulate or dissolved C derived from terrestrial plant tissue) may also shed light on interannual differences beyond the spring burst. The lab study by Grasset et al. (2018) found that while additions of autoOC led to pulses of $F_{{\text{CH}}_{4}}$, alloOC took longer to decompose, and additions led to more gradual but sustained $F_{{\text{CH}}_{4}}$. Thus, the wet spring of 2017 may have loaded the reservoir with slow-burning alloOC, which could partially explain the smaller magnitude of $F_{{\text{CH}}_{4}}$ pulses in 2017 compared to 2018 (Fig. 2).

The impact, or lack thereof, of the spring burst on reservoir-wide cumulative $F_{{\text{CH}}_{4}}$ has implications for the value of higher-resolution measurements. This is analogous to the question of whether the increased complexity of process-based models improves prediction over empirical models (cf. Adams et al., 2013). While the EC monitoring results almost doubled from 2017 to 2018, the hybrid upscaled estimate had only an 11% difference (Table 1, Fig. 5). Furthermore, the cumulative $F_{{\text{CH}}_{4}}$ determined via the lake-wide surveys was closer to the hybrid upscaled estimate than the EC results in 2018 (Fig. 5). Using the recent size-productivity model (DelSontro et al., 2018a) to predict $F_{{\text{CH}}_{4}}$ at Acton Lake based on mean annual chl *a* levels (Eq. 7, Fig. 7) yields estimates of 11.1 and 10.3 Mg CH_4_ m^−2^ h^−1^ for 2017 and 2018, respectively. These values are in the same range as the warm season mean fluxes determined via the hybrid approach for Acton Lake (Table 1). However, the model results contrast with measured results in terms of which year had higher $F_{{\text{CH}}_{4}}$. Furthermore, the model results would overestimate cumulative annual $F_{{\text{CH}}_{4}}$ for Acton Lake as they do not take low wintertime emissions into account.

Sub-annual climatic patterns and productivity dynamics may become more important in understanding and predicting reservoir $F_{{\text{CH}}_{4}}$. Recent research demonstrates how warmer springs have increased the frequency and intensity of cyanobacterial blooms in midwestern US reservoirs over the past two decades (Smucker et al., 2021), and continued warming will likely intensify this phenomenon. There is also a burgeoning body of knowledge that points to the importance of phytoplankton ecology on lake and reservoir CH_4_ production in terms of both the amount (Hartman et al., 2020; McClure et al., 2020; Zhang et al., 2021) and type (Bartosiewicz et al., 2021). Furthermore, the underlying factors that led to the 2018 spring burst at Acton Lake may be more common in the future and have a greater effect on the reservoir CH_4_ budget.

#### 4.2.2 Additional intra-lake variability

**Figure 8.**
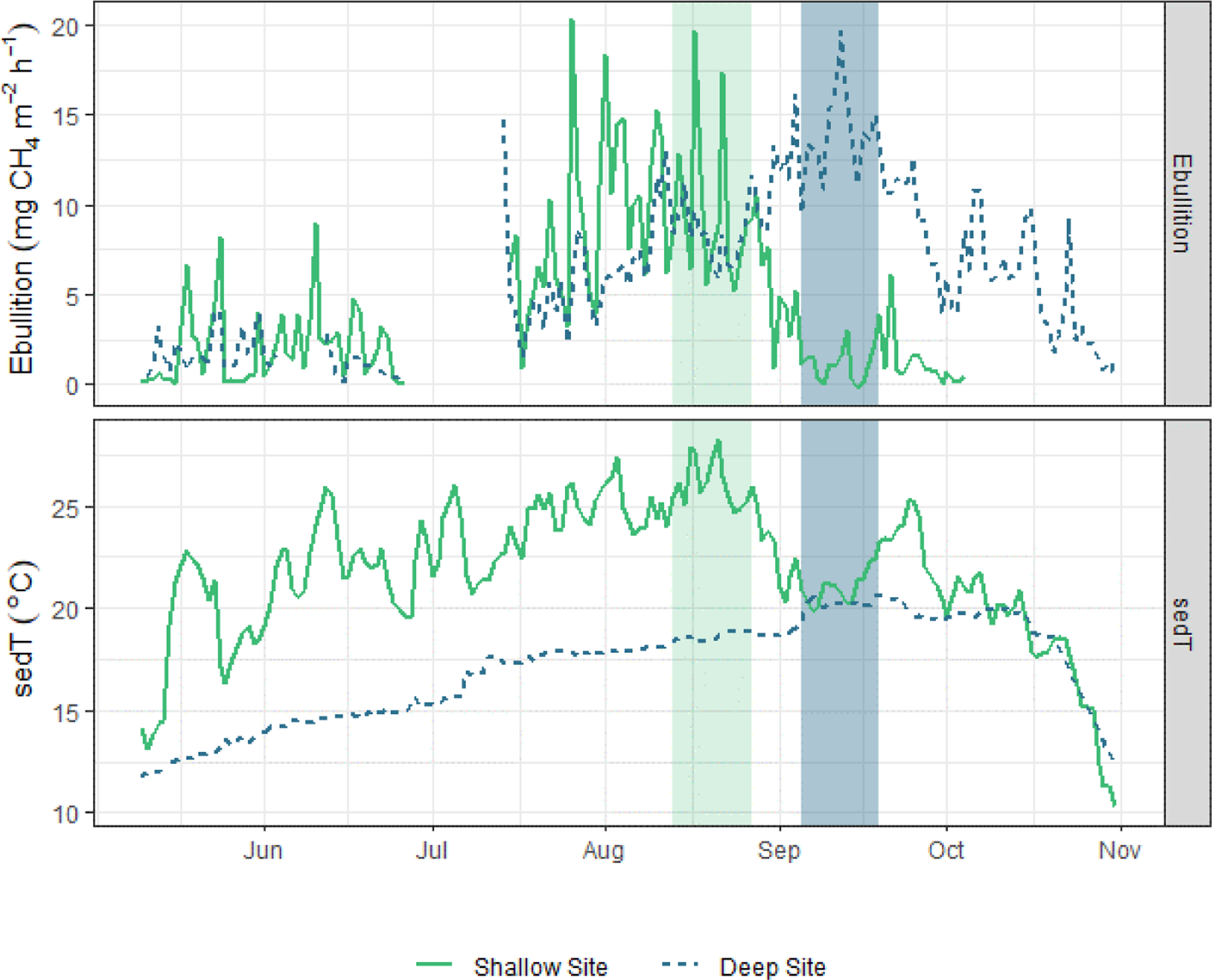
Time series of sedT and ebullition in 2017 at the shallow (solid green line) and deep (dashed blue line) sites. The light grey bar highlights the period of maximum ebullition and sedT at the shallow site; the dark grey bar highlights the corresponding period at the deep site.

Beyond the spring burst, we observed additional patterns of intra-lake spatiotemporal variability in $F_{{\text{CH}}_{4}}$ related to sediment temperature (sedT). Temperature is an important control on metabolic processes such as methanogenesis, but other signals can complicate the relationship between temperature and $F_{{\text{CH}}_{4}}$ at the scale of ecosystem fluxes. Nevertheless, sedT emerged as a key predictor of $F_{{\text{CH}}_{4}}$ in this study. The ANN model used to gap fill the EC monitoring ranked sedT as one of the most important biophysical predictors of $F_{{\text{CH}}_{4}}$ along with absolute static pressure, change in static pressure, and latent heat flux (Fig. 3). A strong indication of the intra-lake patterns in drivers and emissions is that maximum ebullitive $F_{{\text{CH}}_{4}}$ observed by the AFTs coincided with maximum sedT at both the shallow (U-14) and deep (U-12) monitoring sites in 2017 (Fig. 8). This maximum occurs in early August at U-14 vs. mid-September at U-12, a phase shift that reflects the time delay in heat transfer to the deeper sediment. This phase shift could also (speculatively) have been affected by the time delay in nutrient and OC transfer from the inlets. This pattern was not as pronounced in 2018 (Fig. S10) perhaps due to differences in the precipitation regime that affected reservoir metabolism.

We used ecoQ10 and 2DKS threshold analysis to further investigate the role of sediment temperature on regulating $F_{{\text{CH}}_{4}}$ in both the deep and shallow portions of Acton Lake. Both of these quantitative analyses of the relationship between $F_{{\text{CH}}_{4}}$ and sedT yielded statistically significant results (Table 3), and each monitoring method had consistent ecoQ10 values and 2DKS threshold temperatures across the two study years (Table 3, Fig. S11). The EC method had a much lower ecoQ10 value than the AFT sites, the latter of which were comparable to maximum ecoQ10 values reported in other studies (DelSontro et al., 2016). The relatively low ecoQ10 value for the EC method may be due to the different temperature response of ebullitive vs. diffusive emission pathways or to a spatial mismatch between the measured sedT and the EC flux footprint. For these reasons, we focus on the AFT sites in interpreting the ecoQ10 and threshold temperature results in terms of intra-lake spatial variability. The ecoQ10 values indicate a stronger relationship between sedT and ebullitive $F_{{\text{CH}}_{4}}$ at the shallow site than the deep site. Despite a greater ecoQ10 value, ebullitive $F_{{\text{CH}}_{4}}$ at the shallow site did not respond to warming in the spring until water temperatures reached a threshold of ~ 22.5 °C, whereas ebullitive $F_{{\text{CH}}_{4}}$ at the deep site responded to warming at a much lower temperature threshold (13–18 °C; Table 3). Furthermore, mean ebullitive $F_{{\text{CH}}_{4}}$ was very similar between the two sites (Table 1) despite a 6°C difference in maximum sed iment temperature. These patterns suggest that methanogens at the deep site may be better adapted to the consistently cooler conditions found in the hypolimnion of Acton Lake, which has important implications for predictive models employing ecoQ10 or threshold values to parameterize $F_{{\text{CH}}_{4}}$ as a function of sedT. Alternatively, the differences in temperature sensitivity between the deep and shallow site may reflect differences in substrate quality and/or quantity related to spatial patterns in sedimentation and productivity (Berberich et al., 2019). Regardless of the underlying mechanism, these patterns illustrate strong spatial patterning in CH_4_ biogeochemistry within this 2.4 km^2^ reservoir.

## 5 Conclusions

In this study we investigated temporal patterns and biophysical drivers of CH_4_ fluxes from a eutrophic temperate reservoir using multiple methods including eddy covariance. Sediment temperature and the overlying static pressure were the most important biophysical drivers of $F_{{\text{CH}}_{4}}$ per the ANN model results. Water chemistry and chl *a* measurements indicate that the spring burst of elevated $F_{{\text{CH}}_{4}}$ coincided with a phytoplankton bloom. Comparing the two observation years indicated that the climatic conditions of precipitation and temperature were more conducive to a phytoplankton bloom in 2018 than 2017. In contrast to previous studies, we saw a weak positive correlation between $F_{{\text{CH}}_{4}}$ and reservoir depth, we did not find a strong relationship between $F_{{\text{CH}}_{4}}$ and underwater turbulence, nor did we observe consistent diurnal patterns in $F_{{\text{CH}}_{4}}$.

We found that Acton Lake had cumulative annual CH_4_ areal emissions of 45.6 ± 8.3 and 51.4 ± 4.3 g CH_4_ m^−2^ in 2017 and 2018, respectively. These levels of emissions place Acton Lake in the upper quartile of emission rates reported from reservoirs (Deemer et al., 2016), further supporting the concept that highly productive midlatitude reservoirs can have higher-magnitude CH_4_ emission rates than would be predicted by age and latitude alone (DelSontro et al., 2018a). A spring burst of $F_{{\text{CH}}_{4}}$ observed over a 2-week period in 2018 but not 2017 accounted for 59 % of the difference in cumulative emissions between years. This difference between consecutive years highlights the importance of multi-year studies (cf. Room et al., 2014) and the importance of characterizing temporal variability in open-water systems, which Williamson et al. (2020) illustrated exceeded spatial variability for several physical, chemical, and biological metrics.

The EC technique holds much promise for improving our understanding of the biophysical drivers of gaseous fluxes, with a few caveats. In addition to the pseudo-continuous temporal coverage, the EC measurement footprint encompasses a much larger area than traditional gas flux measurement techniques (e.g., dissolved gas sampling, chambers, inverted funnel traps), increasing the likelihood of integrating fluxes over a distribution of hot spots. However, care must be taken in the siting, quality control, and interpretation of results. The authors reemphasize the recommendation given by Vesala et al. (2012): for best results, close collaboration is needed between biometeorologists and limnologists to understand what is going on both above and below the water. For future studies of reservoir $F_{{\text{CH}}_{4}}$ using EC, we recommend siting the monitoring tower in the area of the reservoir with the highest variability in CH_4_ emissions, likely near the inlet, and setting up multiple AFTs across the reach of the reservoir to constrain spatial patterns. Future studies that incorporate more direct measurements of phytoplankton dynamics would also be useful to improve our understanding of drivers of CH_4_ production and emission that may be more common with future warmer springs and extremes in precipitation patterns.

The EC results in this study further our understanding of the interaction between precipitation, sediment temperature, algal productivity levels, and $F_{{\text{CH}}_{4}}$. This study adds to our understanding of open-water flux processes at appropriate spatial and temporal scales while highlighting a way to present and compare EC and whole-reservoir survey data in appropriate contexts.

## Supplementary Material

Supplement1

## Figures and Tables

**Table 1.** Seasonal methane fluxes reported as mean fluxes and cumulative areal emissions from Acton Lake characterized by different measurement techniques. The eddy covariance method measures total (diffusive + ebullitive + other) fluxes.

		Warm season* mean flux (mg CH_4_ m^−2^ h^−1^)			Cumulative annual emissions (g CH_4_ m^−2^)

	Observation type	Diffusive	Ebullitive	Total	Total
2017	Eddy covariance	–	–	9.73 ± 0.67	40.7 ± 5.9
	Shallow site	3.2	4.47 ± 0.63	7.67 ± 0.63	29.3 ± 2.2
	Deep site	0.89	5.76 ± 0.54	6.67 ± 0.54	29.0 ± 2.0
	Lake surveys	1.28 ± 0.52	8.71 ± 6.1	9.98 ± 6.2	37.4 ± 5.6
	Hybrid upscaled	–	–	10.3 ± 1.9	45.6 ± 8.3

2018	Eddy covariance	–	–	17.5 ± 0.38	71.4 ± 4.2
	Shallow site	3.55	5.68 ± 0.11	9.74 ± 0.11	41.9 ± 0.36
	Deep site	0.96	6.65 ± 0.05	7.57 ± 0.05	30.8 ± 0.25
	Lake surveys	1.87 ± 1.2	11.1 ± 6.1	13.0 ± 6.6	49.2 ± 3.7
	Hybrid upscaled	–	–	12.9 ± 0.96	51.4 ± 4.3

* “Warm season” is defined as 1 May–30 September.

**Table 2.** Dissolved nutrient and carbon data for the inflow and outflow during the study period, reported as the mean of weekly samples taken between April and October and as the value measured for the week of the 2018 spring burst (24 May–4 June). Dissolved nutrient data include total nitrogen (TN), ammonium (NH_4_), nitrate (NO_3_), total phosphorus (TP), and soluble reactive phosphorus (SRP). Dissolved carbon was measured as particulate organic carbon (POC).

	2017				2018			
	
	Mean		Spring burst		Mean		Spring burst	
	
Analyte (units)	Inflow	Outflow	Inflow	Outflow	Inflow	Outflow	Inflow	Outflow
TN (mg N L^−1^)	5.69	5.30	8.27	8.12	2.05	1.78	3.39	3.03
NH_4_ (mg N L^−1^)	0.05	0.07	0.02	0.02	0.05	0.05	0.17	0.07
NO_3_ (mg N L^−1^)	0.97	0.89	1.69	1.62	0.25	0.22	0.47	0.43
TP (µg P L^−1^)	115	99.9	98.6	76.6	141	80.4	254	110
SRP (µg L^−1^)	20.2	24.4	2.66	5.35	11.5	9.69	15.7	2.81
POC (mg L^−1^)	3.53	2.69	3.42	2.96	4.09	2.74	4.48	3.06

**Table 3.** Summary statistics describing the relationship between $F_{{\text{CH}}_{4}}$ and sediment temperature per the ecoQ10 analysis and the two-dimensional Kolmogorov–Smirnov test (2DKS) threshold analysis.

		Eddy covariance	AFT shallow	AFT deep
ecoQ10	2017 value	6.96	35.1	30.4
	2017 *R*^2^	0.85	0.48	0.60
	2018 value	5.64	35.8	30.7
	2018 *R*^2^	0.83	0.85	0.38

Threshold (2DKS)	2017 sedT threshold	14.1	22.2	17.9
	2017 test statistic	0.226	0.166	0.204
	2018 sedT threshold	17.4	23.0	13.3
	2018 test statistic	0.234	0.190	0.138
