# Supplementary material for: Temporal trends in methane emissions from a small eutrophic reservoir: the key role of a spring burst
Source: Biogeosciences. Author manuscript; Available in PMC 2022 Sep 30. (PMC8815417; doi:10.5194/bg-18-5291-2021)
Supplement: Supplement1 [file NIHMS1745327-supplement-Supplement1.pdf]

Supplement of Biogeosciences, 18, 5291–5311, 2021
https://doi.org/10.5194/bg-18-5291-2021-supplement
© Author(s) 2021. CC BY 4.0 License.

Biogeosciences
Open Access

Supplement of

Temporal trends in methane emissions from a small eutrophic reservoir: the key role of a spring burst

Sarah Waldo et al.

Correspondence to: Sarah Waldo (sarahrwaldo@gmail.com)

The copyright of individual parts of the supplement might differ from the article licence.

Table S1: Measurement methods summary

Method	*Flux Measured*	*Spatial Coverage*	*Frequency*	*Use*
Eddy Covariance (EC)	total net	~100s m^2, north sector of the lake	pseudo-continuous, 30-min timestep	• annual budgets<br>• diurnal patterns<br>• biophysical drivers: ANN, Q10, 2DKS
Active Funnel Traps (AFT)	ebullition	0.3 m^2, two locations	pseudo-continuous, 30-min timestep	• annual budgets<br>• diurnal patterns<br>• biophysical drivers: Q10, 2DKS
Flux Chamber	diffusion	0.2 m^2 per site	2 sites bi-weekly; 15 sites sampled during 6 GRTS surveys	• annual budgets<br>• emission pathway relative importance
Passive Funnel Traps	ebullition	0.3 m^2	15 sites sampled during 6 GRTS surveys	• annual budgets<br>• spatial patterns<br>• emission pathway relative importance

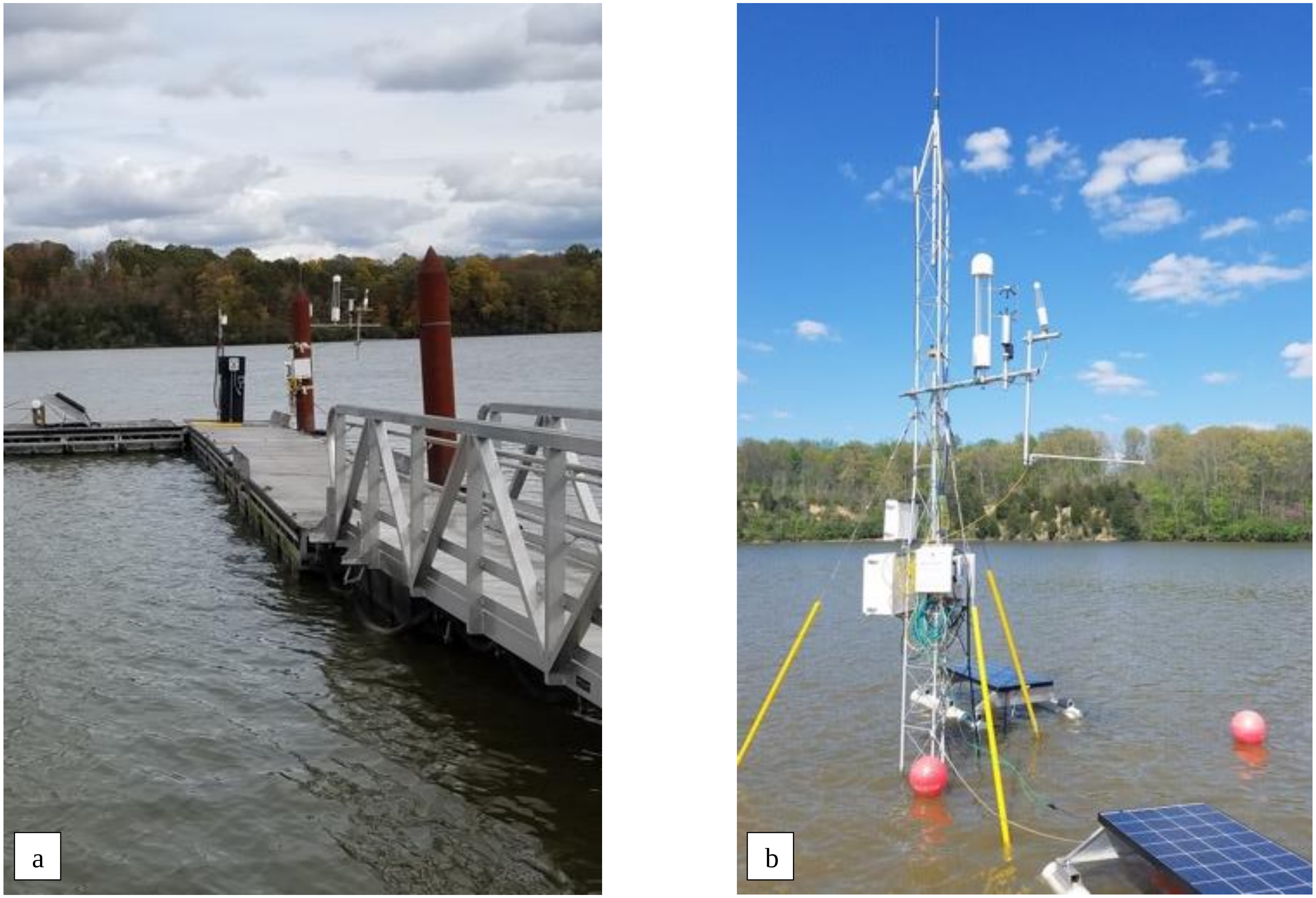
a
b

Figure S1: Pictures of eddy covariance measurement locations. Panel a (left): site S-1, dock location for February 2017 – April 2018 monitoring. Panel b (right): site S-2, open-water location for May – November 2018 monitoring.

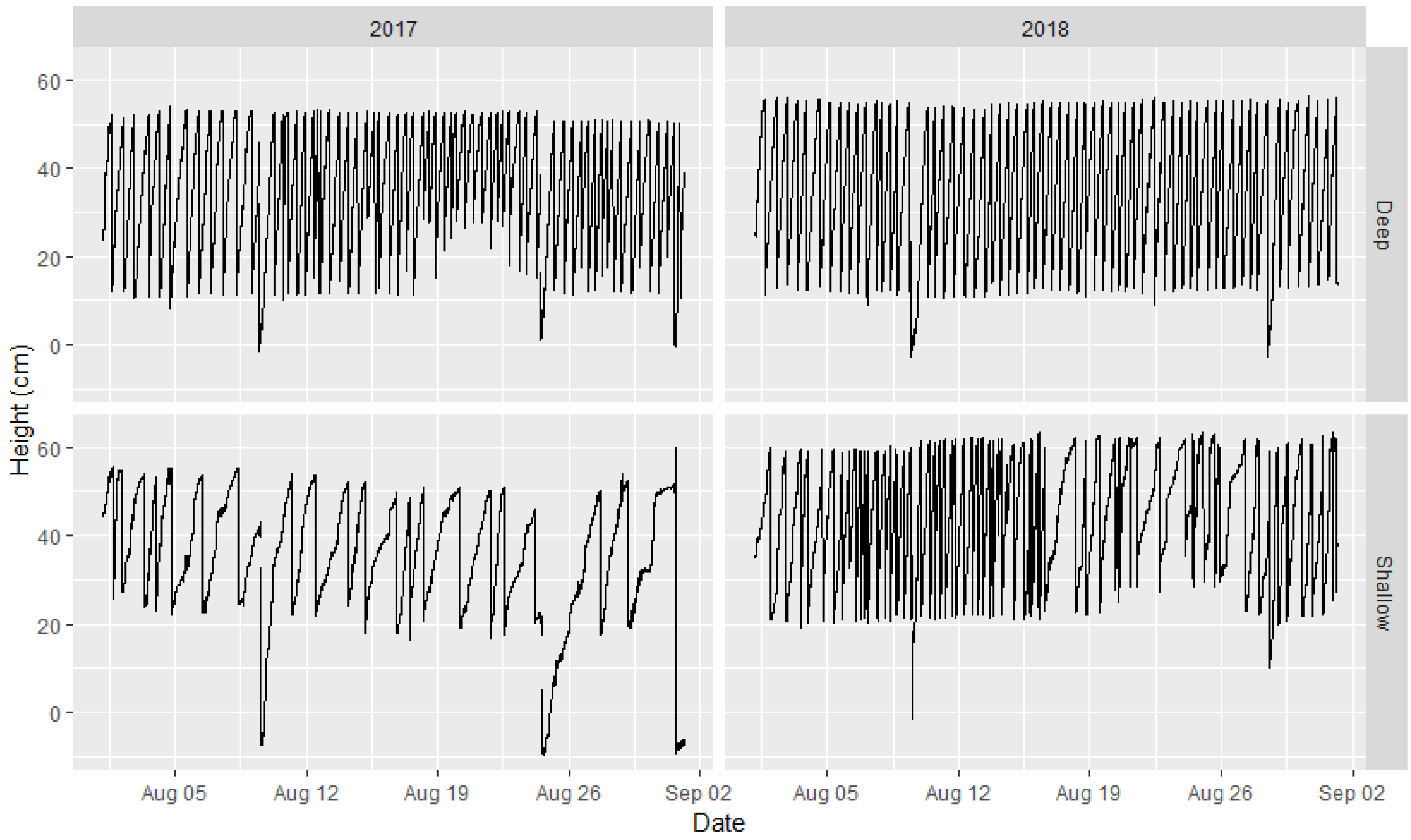

Figure S2: Time series of the raw active trap volume measurements from the two monitoring sites: the deep site (U-12, top), and the shallow site (U-14, bottom).

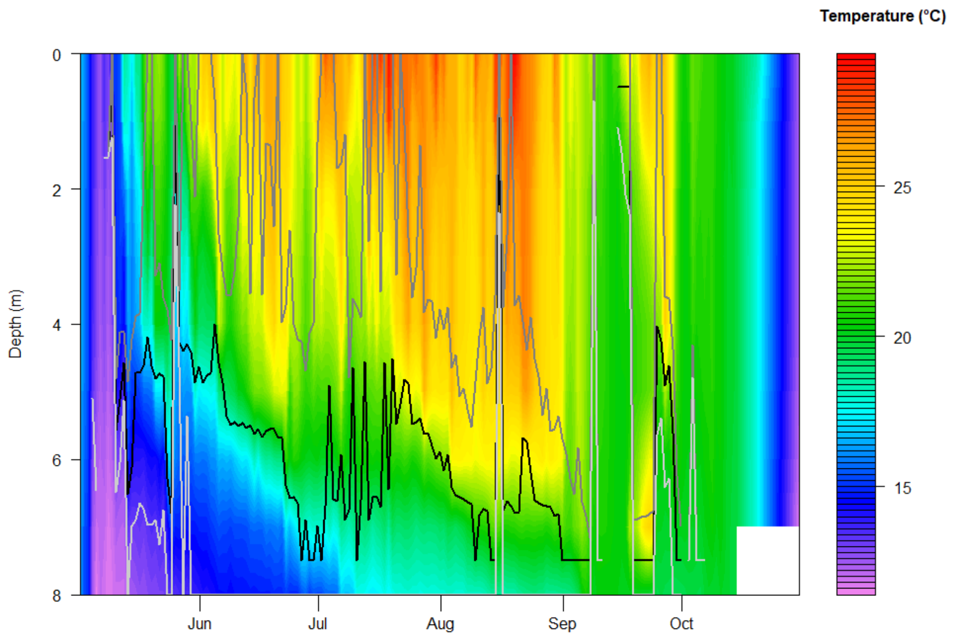

2017

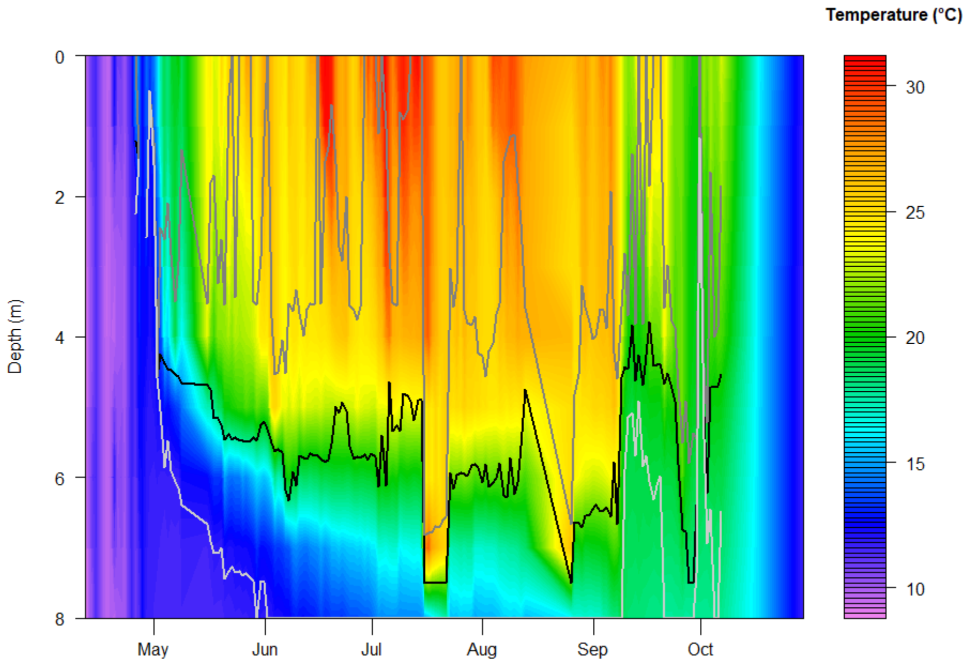

2018

Figure S3: Time series of the water temperature profile including thermocline depth (black line) in 2017 (top) and 2018 (bottom) at the U-14 deep site in Acton Lake.

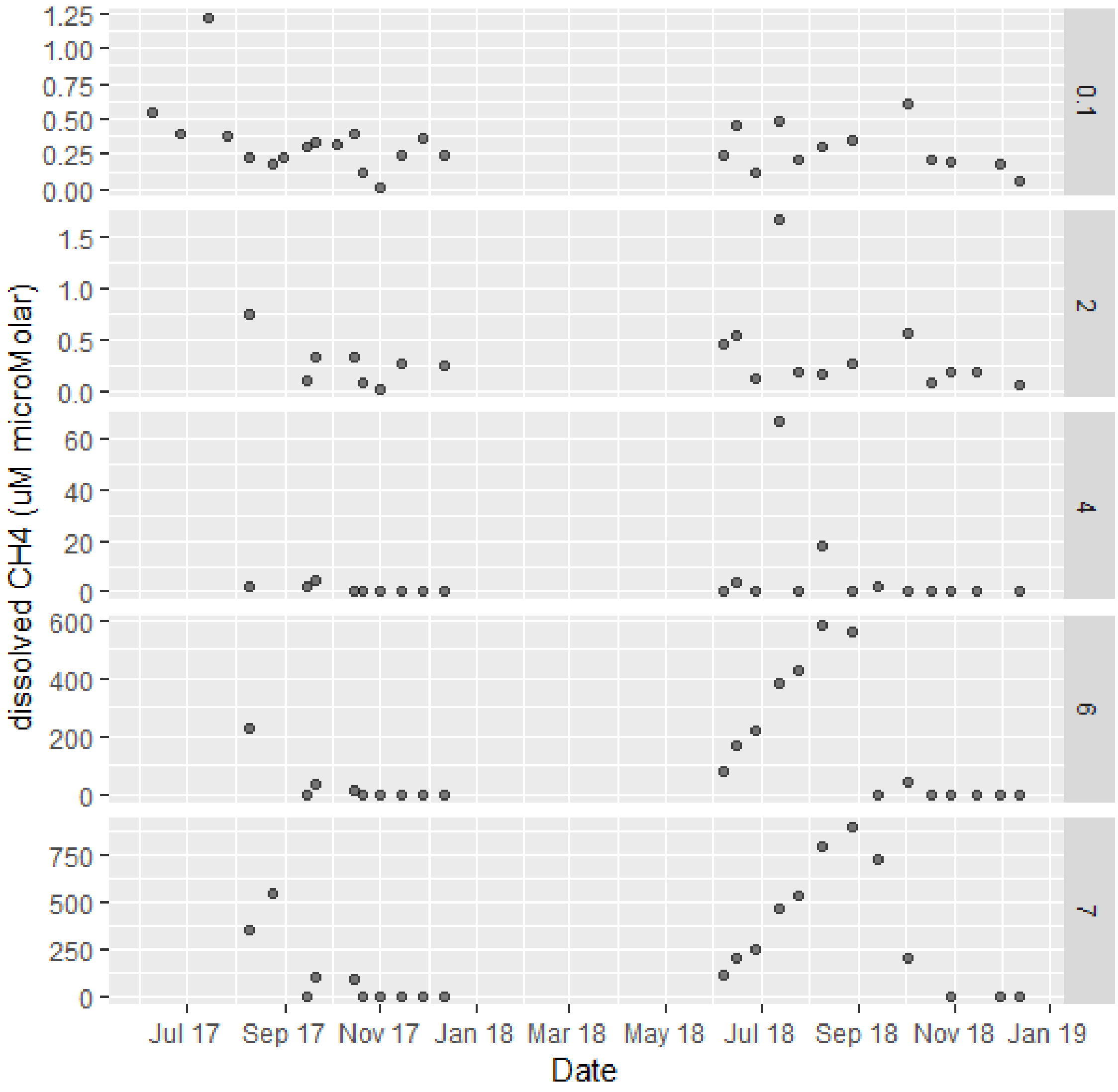

Figure S4: Time series of the depth profile of dissolved CH_4 (uM) at the deep site (U-14) over the 2017-2018 study period. Numbers on the right panels indicate depth in m below surface.

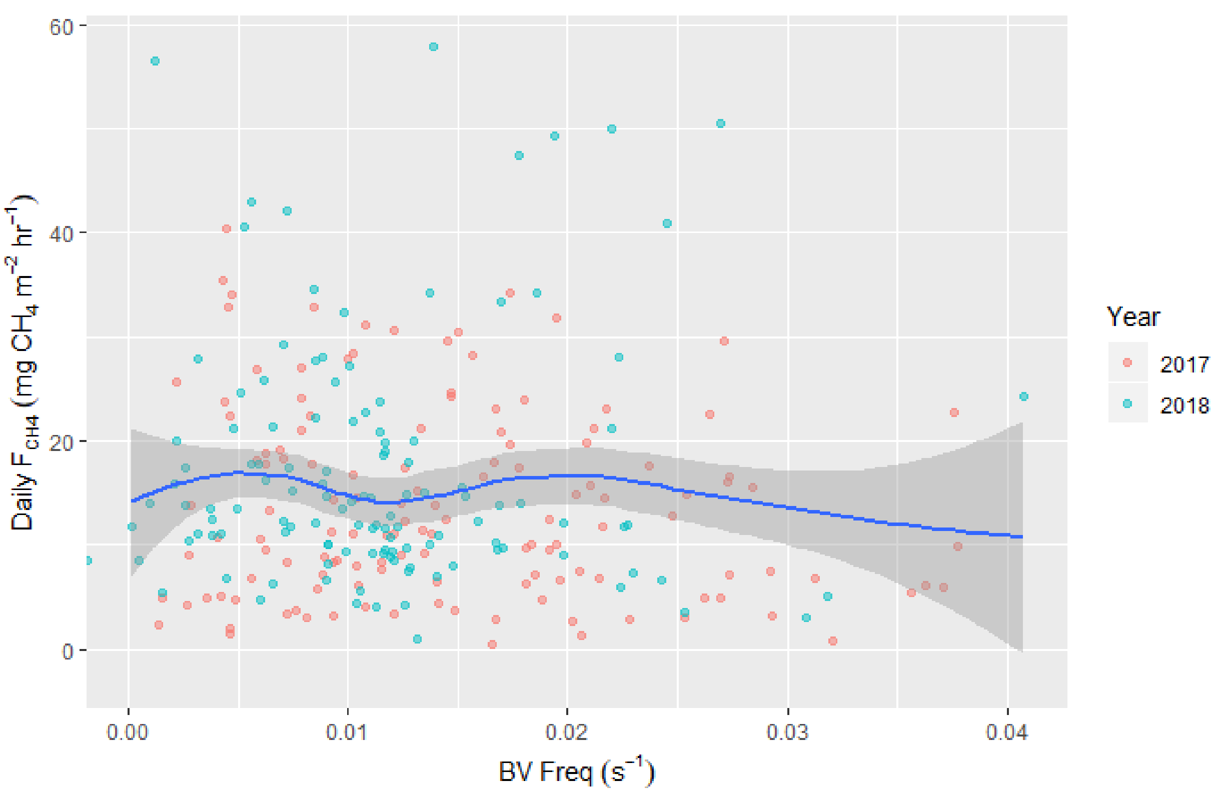

Figure S5: Scatterplot of daily F_{CH4} as a function of the Brunt-Väisälä frequency, an indicator of underwater turbulence.

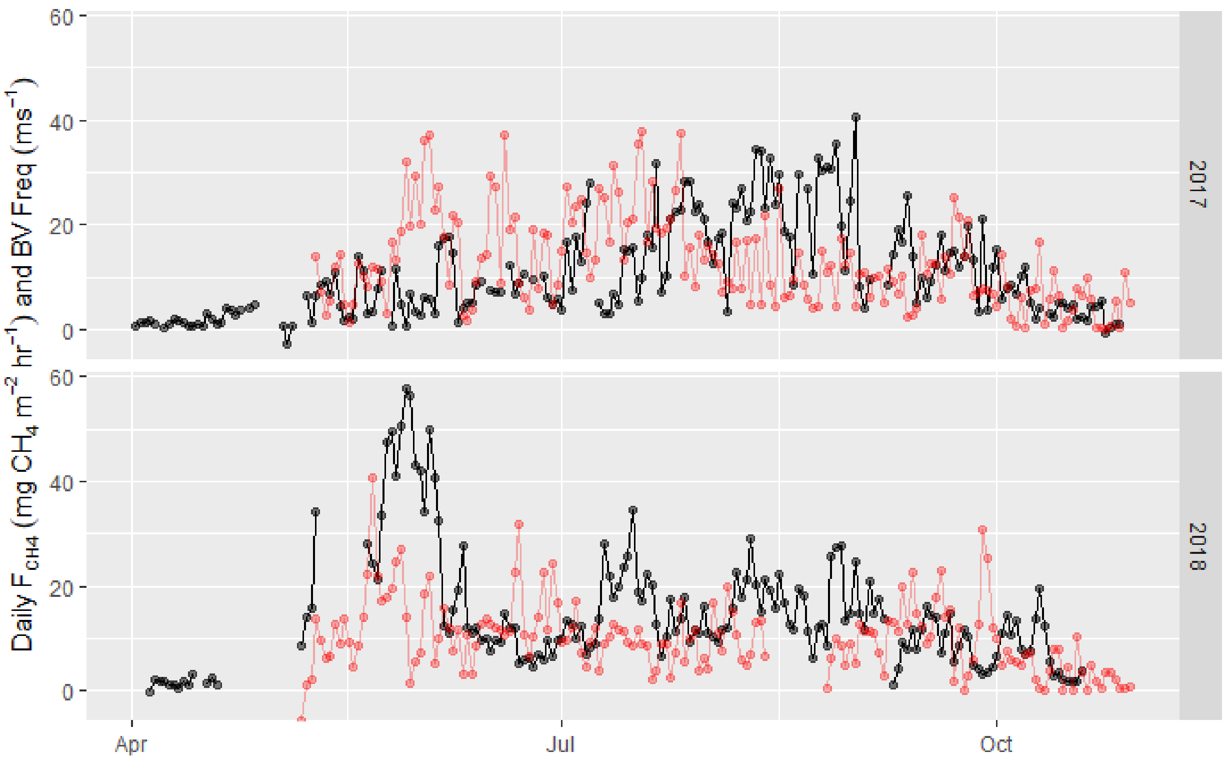

Figure S6: Time series of daily F_{CH4} (black) and Brunt-Väisälä frequency (red) plotted on the same axes.

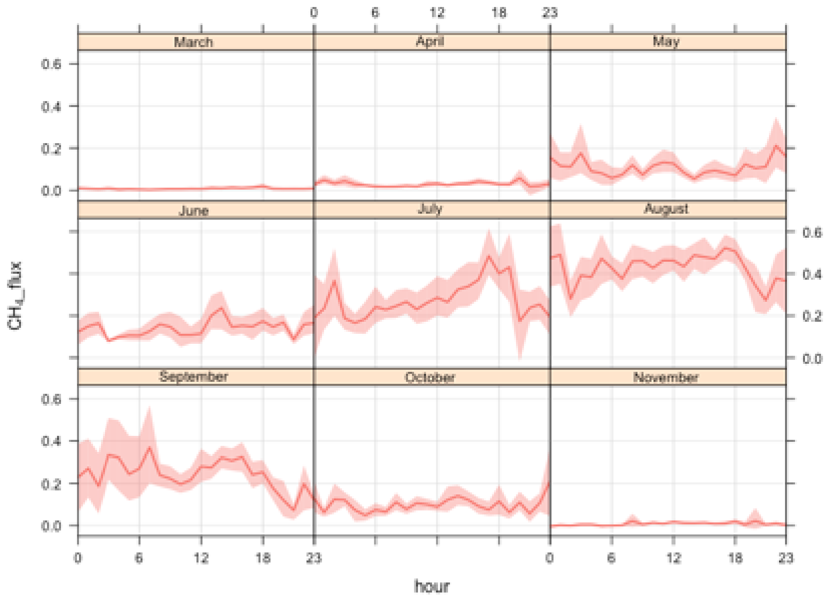

2017

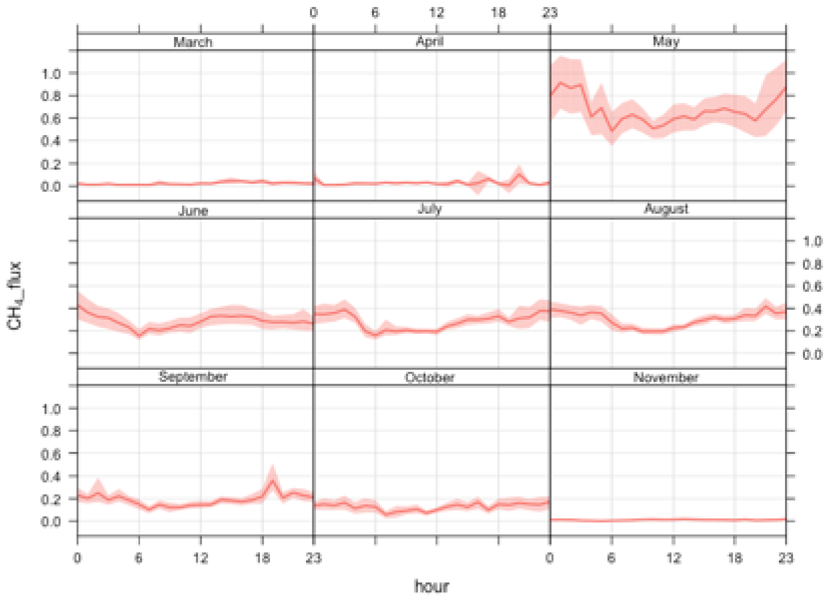

2018

Figure S7: Diurnal F_{CH4} (umol m-2 h-1) aggregated over monthly time periods from March-Nov for 2017 (left) and 2018 (right).

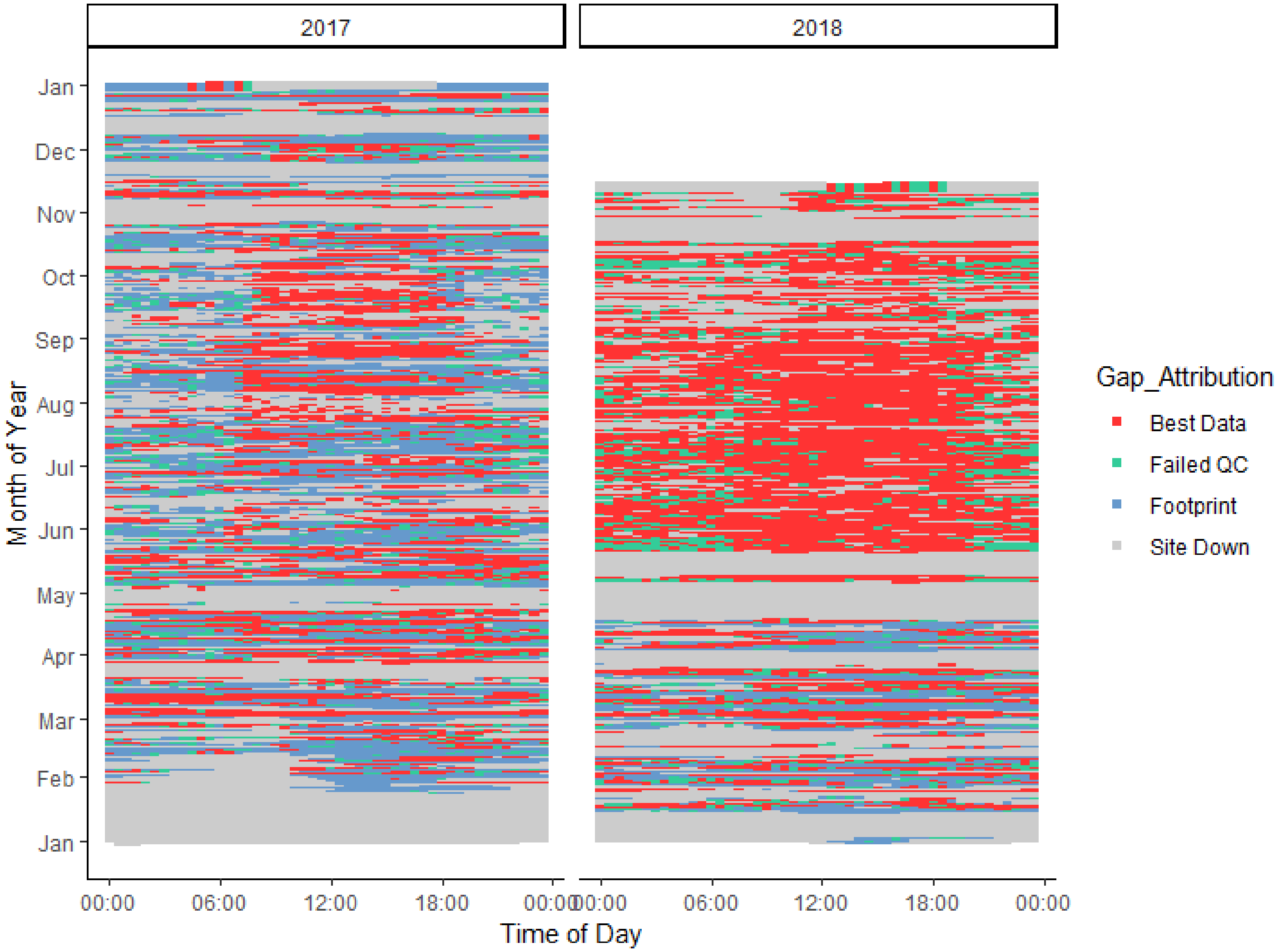

Figure S8: Eddy covariance gap attribution and distribution for 2017 and 2018. Each pixel represents one 30-minute flux measurement period.

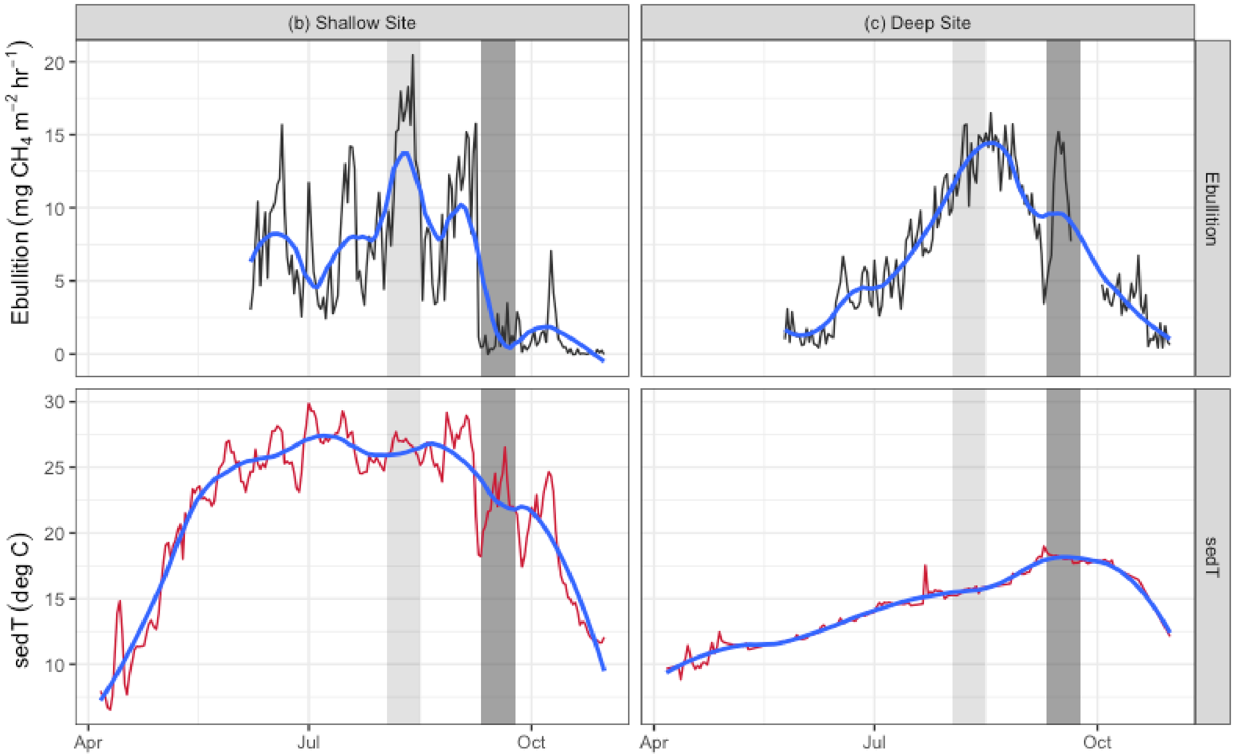

Figure S9: Time series of sedT and ebullition in 2018 at the shallow (a, U-14) and deep (b, U-12) sites. This year did not display the offset relationship between maximum sedT and maximum ebullition observed in 2017 (Fig. 10).

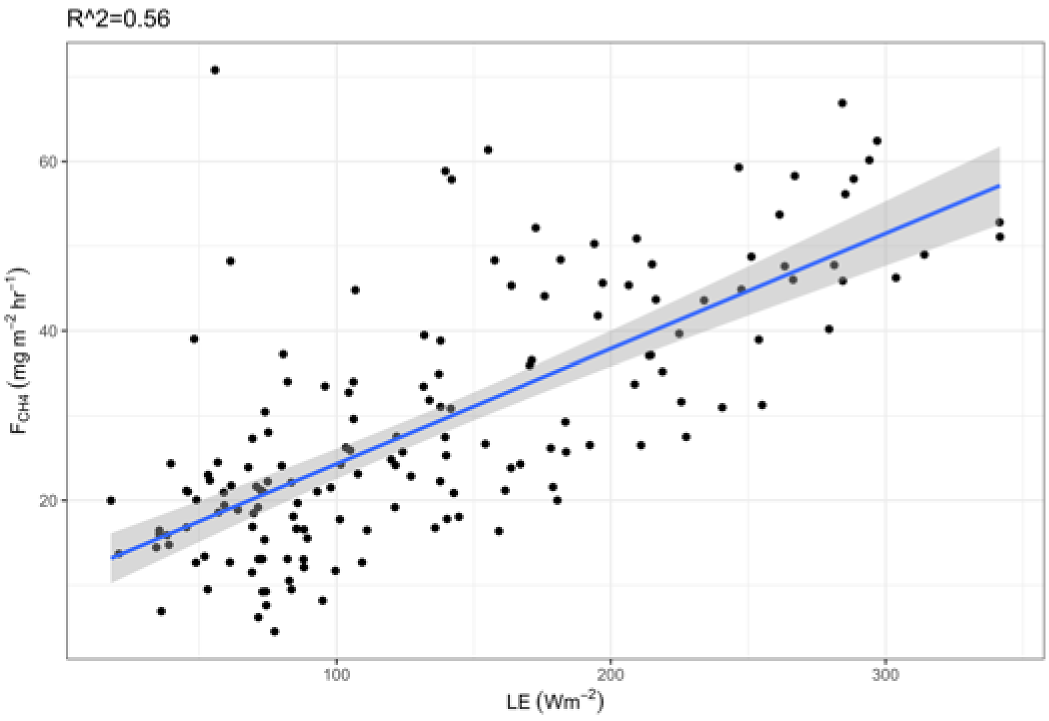

Figure S10: 30-minute methane fluxes as a function of latent heat flux (LE) for several-day period pre- and post-spring burst in 2018 (depicted in Fig 12 (a)). Significant at $p<0.001$.

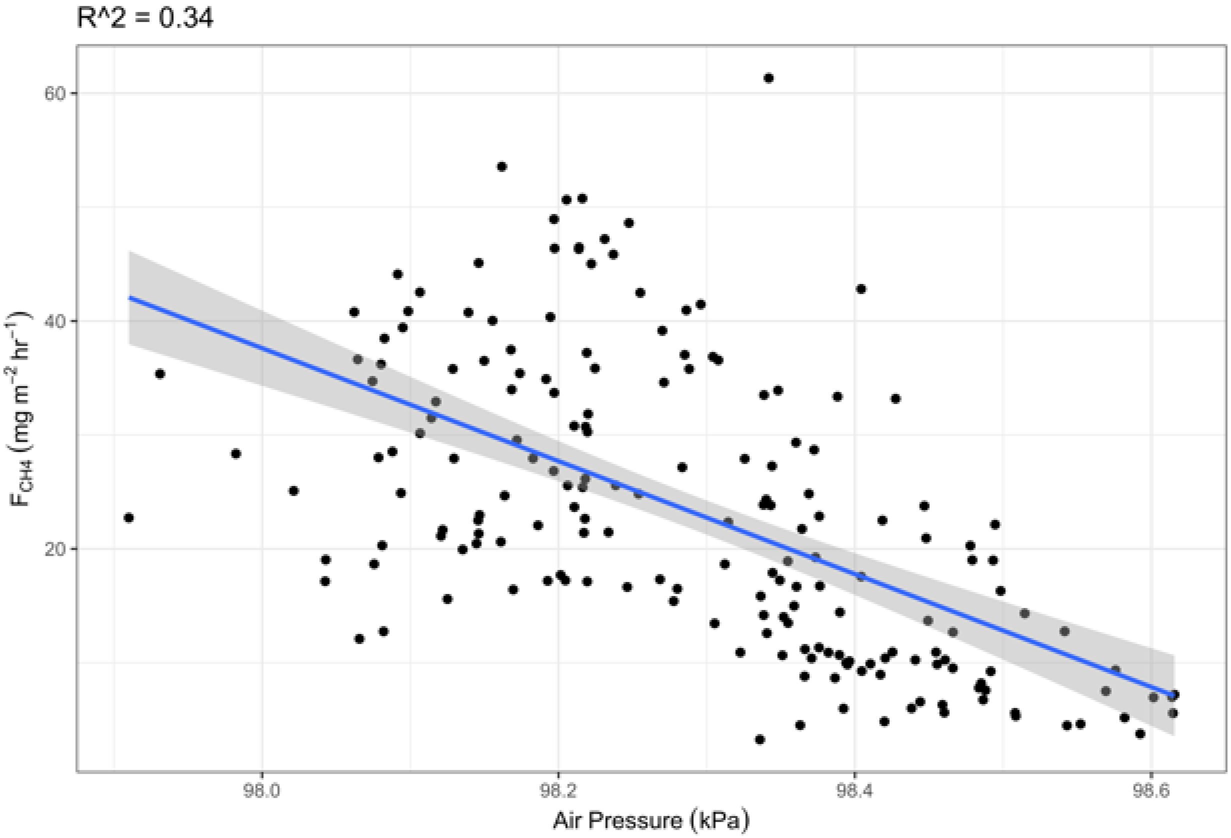

Figure S11: 30-minute methane flux as a function of air pressure for the several-day period depicted in Fig 12 (b). Significant at p<0.001.
